# Preparation and Biological Activity of Lignin–Silver Hybrid Nanoparticles

**DOI:** 10.1021/acsomega.4c08117

**Published:** 2024-11-20

**Authors:** Dominik Maršík, Matěj Danda, Jaroslav Otta, Petter P. Thoresen, Olga Mat́átková, Ulrika Rova, Paul Christakopoulos, Leonidas Matsakas, Jan Masák

**Affiliations:** † Department of Biotechnology, University of Chemistry and Technology, Prague 166 28, Czech Republic; ‡ Department of Physics and Measurements, University of Chemistry and Technology, Prague 166 28, Czech Republic; § Biochemical Process Engineering, Division of Chemical Engineering, Department of Civil, Environmental, and Natural Resources, Luleå University of Technology, Luleå 971 87, Sweden

## Abstract

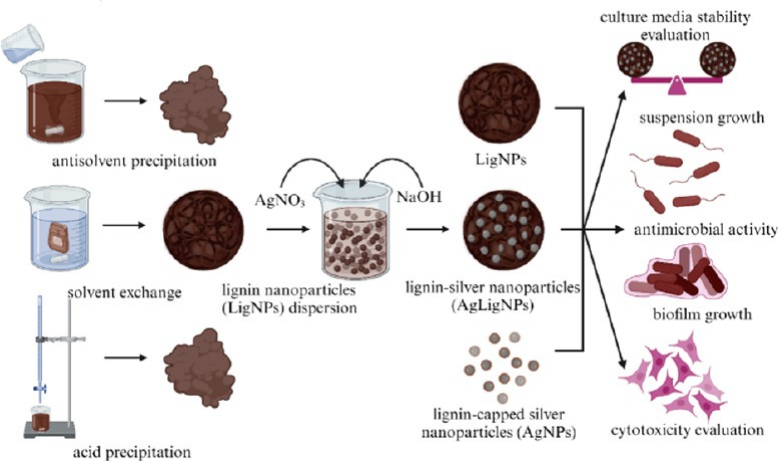

Silver nanoparticles (AgNPs) are excellent antimicrobial agents and promising candidates for preventing or treating bacterial infections caused by antibiotic resistant strains. However, their increasing use in commercial products raises concerns about their environmental impact. In addition, traditional physicochemical approaches often involve harmful agents and excessive energy consumption, resulting in AgNPs with short-term colloidal stability and silver ion leaching. To address these issues, we designed stable hybrid lignin–silver nanoparticles (AgLigNPs) intended to effectively hit bacterial envelopes as a main antimicrobial target. The lignin nanoparticles (LigNPs), serving as a reducing and stabilizing agent for AgNPs, have a median size of 256 nm and a circularity of 0.985. These LigNPs were prepared using the dialysis solvent exchange method, producing spherical particles stable under alkaline conditions and featuring reducing groups oriented toward a wrinkled surface, facilitating AgNPs synthesis and attachment. Maximum accumulation of silver on the LigNP surface was observed at a mass reaction ratio m_Ag_:m_Lig_ of 0.25, at pH 11. The AgLigNPs completely inhibited suspension growth and reduced biofilm development by 50% in three tested strains of *Pseudomonas aeruginosa* at a concentration of 80/9.5 (lignin/silver) mg L^–1^. Compared to unattached AgNPs, AgLigNPs required two to eight times lower silver concentrations to achieve complete inhibition. Additionally, our silver-containing nanosystems were effective against bacteria at safe concentrations in HEK-293 and HaCaT tissue cultures. Stability experiments revealed that the nanosystems tend to aggregate in media used for bacterial cell cultures but remain stable in media used for tissue cultures. In all tested media, the nanoparticles retained their integrity, and the presence of lignin facilitated the prevention of silver ions from leaching. Overall, our data demonstrate the suitability of AgLigNPs for further valorization in the biomedical sector.

## Introduction

With the rising antibiotic resistance among many bacterial species, there is an urgent societal need to explore alternative methods for preventing bacterial infections.^1^ In this regard, metal nanoparticles, especially silver nanoparticles (AgNPs), have been widely studied due to their considerable antimicrobial activity in nanoform.^2^ Although the exact mechanism of action of AgNPs is not fully understood,^3^ it involves nonspecific effects, including interaction with the bacterial membranes and its subsequent destabilization, leading to the disruption of vital bacterial processes. Additionally, it is generally accepted that AgNPs can penetrate bacterial cells and disrupt internal components^4−6^ and in the presence of Ag^+^ ions, a duplex comprising the imidazole–Ag^+^–imidazole base pairs is formed within the DNA structure.^7^ However, for Gram-negative bacteria, the primary target of AgNPs is the plasma membrane and its associated cellular processes.^8,9^ This susceptibility is significantly influenced by factors such as nanoparticle size, shape,^10−12^ and the surrounding medium conditions.^13^ Additionally, the effect of AgNPs depends on their capping agent,^14^ which influences the stability of their silver cores. The release of silver ions, which are toxic to both bacterial and animal cells, impacts their further use.^15^ Moreover, the silver ions’ release is a matter of controversy owing to the environmental risk associated with the extensive use of AgNP-based commercial products, which constitute the most commonly utilized nanomaterials.^16^ Silver ions released into the environment can threaten aquatic organisms,^17^ making it essential to develop nanosystems that maximize the antimicrobial effect while minimizing the overuse of silver in commercial products. To enhance the stability of AgNPs, it is advantageous to prepare them by using biological methods, which envelope the silver cores with complex substances that can impart additional properties, such as enhanced antimicrobial effects.^18−20^ Nanoparticles prepared using these biological methods are often modified with complex phenolic compounds, which are responsible for both the reduction of the precursor and the stability of the silver core.^21^

Lignin, a widely available and renewable polymer with a polyphenolic framework,^22^ is being explored for valorization into valuable products.^23^ Due to its low cytotoxicity^24,25^ and natural origin,^26^ lignin is considered a safe polymer that should not pose an environmental risk as a part of hybrid nanoparticles. Furthermore, lignin in its nanoform has demonstrated antimicrobial effects by generating reactive oxygen species (ROS) and destabilizing bacterial membranes.^27,28^ The complex polyphenol structure and its nonspecific effects make it difficult for bacteria to develop resistance. Additionally, lignin’s polyphenolic structure facilitates noncovalent interactions with proteins in water, promoting direct contact between silver and membrane surface proteins.^29^ These properties suggest that LigNPs could synergistically enhance the effect of AgNPs, which, despite their nonspecific antimicrobial mechanism, have already been reported to potentially lead to resistance development.^30−32^ Additionally, for many applications, it is often crucial to convert lignin into an aqueous solution with a neutral pH. This is typically not possible for the raw lignin without chemical modification.^33^ However, in the form of NPs, lignin can form a stable colloidal dispersion in aqueous solutions.^34,35^ This increases the surface area-to-volume ratio and the number of particles available for interactions, thereby generally enhancing the system’s reactivity.^36^ For these reasons, we proposed the synthesis of a lignin carrier with AgNPs accumulated on its surface to effectively target bacterial membranes by increasing local concentration.^37^ Our focus is on the synthesis of spherical, homogeneous nanoparticles, which are desirable for the medical and healthcare sector.^38^ Previous studies have successfully modified LigNPs with noble metals, including tellurium,^39^ gold,^40^ or palladium.^41^ In a recent study, silver was also used, where the reduction of the Ag^+^ precursor on the surface of LigNPs was accelerated using UV light.^42,43^ However, this approach did not result in strong coverage of the LigNP surface, which we identified as one of the crucial conditions for maximizing the antibacterial efficiency of hybrid nanoparticles.

In this study, we aim to demonstrate the effect of Ag accumulation on LigNPs in comparison to NPs made up of pure Ag, which were prepared and characterized in our previous work^44^ through the reduction process of the silver precursor by dissolved lignin, acting as both a reducing and capping agent, while maintaining the green character of the synthesis.

The antimicrobial efficacy was evaluated against the Gram-negative bacterium *Pseudomonas aeruginosa*, known for forming resilient biofilms^45^ and serving as a model organism for biofilm formation.^46^ This opportunistic pathogen causes a range of acute and chronic infections, including pneumonia, meningitis, abscesses, skin and soft tissue infections, urinary tract infections, bone and joint infections, and bacteremia.^47−50^ At-risk groups include immunocompromised patients, such as those with chronic obstructive pulmonary disease, cystic fibrosis, trauma, burns, ventilator-associated pneumonia, cancer, HIV, and diabetes.^51−54^ The growing incidence of resistant strains of *P. aeruginosa* is a significant concern,^55^ highlighted by the World Health Organization (WHO).^56^ The design of silver-based composite materials could potentially offer alternative treatments or enhance infection prevention, thereby supporting efforts to address the increasing prevalence of antibiotic-resistant bacterial strains. Furthermore, to reliably assess the antimicrobial activity of our nanosystems, we conducted an evaluation of stability within the culture media. Finally, we complement our findings with cytotoxicity evaluations of our nanosystems to outline the possibility of another valorization. For this purpose, we selected two cell lines: HEK-293, derived from embryonic kidney cells, as a model to assess overall cytotoxicity in human cells, and HaCaT, an immortalized human epidermal keratinocyte line, representing epidermal tissue, which is pertinent to the potential application of lignin–silver hybrid nanoparticles.^57−59^

## Results and Discussion

### Fabrication and Characterization of LigNPs

#### Influence of the Preparation Method on LigNP Morphology

**Figure 1:**
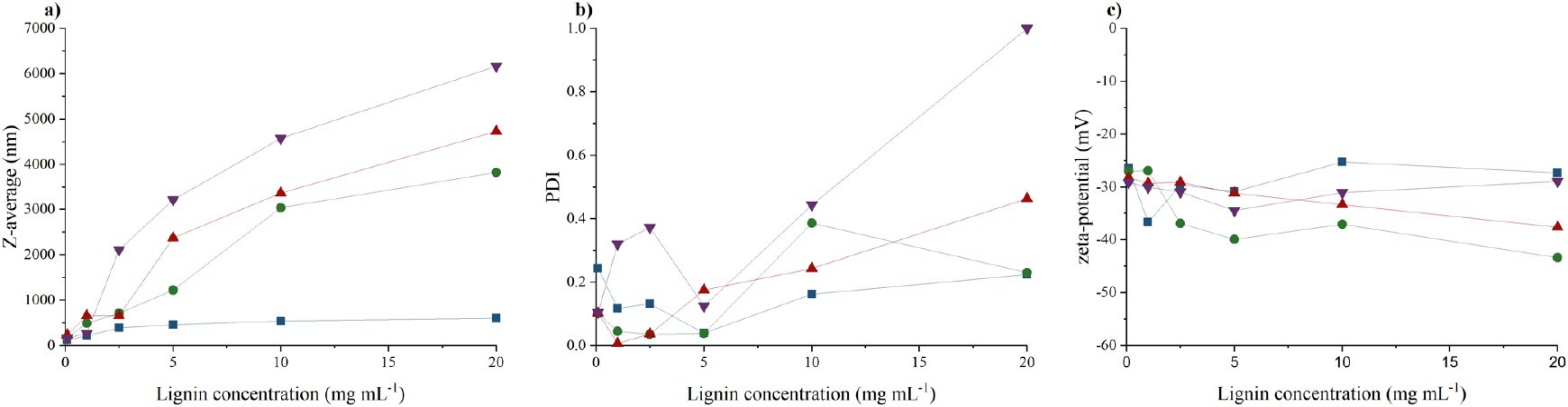
Influence of input lignin concentration on LigNPs: (a) intensity-weighted mean hydrodynamic size (*Z*-average), (b) polydispersity index (PDI), and (c) zeta-potential values prepared using three distinct methods: antisolvent precipitation (blue filled square), dialysis solvent exchange 14,000 MWCO (green filled circle), dialysis solvent exchange 3,500 MWCO (red filled triangle), and acid precipitation (purple down-pointing filled triangle).

To introduce the lignin–silver hybrid nanoparticles (AgLigNPs) synthesis, the initial focus was on the choice of method for the LigNPs’ preparation. Lignin extracted by organosolv pretreatment of beech sawdust was converted into particles by using three distinct methods: antisolvent precipitation, solvent exchange, and acid precipitation. The aim was to obtain LigNPs with uniform and spherical morphology stabilized in water and across a broad pH range. The variable factor in LigNPs’ preparation through the selected methods was the initial concentration of lignin. As depicted in Figure 1, the hydrodynamic diameter generally correlated with increasing lignin concentration. The input lignin concentration played a decisive role in the size of the dispersed phase, linked to an increasing particle’s hydrodynamic diameter and higher probability of aggregate formation. The impact on dispersion phase size resulting from antisolvent precipitation was minor, consistent with the observations of Matsakas et al.^60^ This is a consequence of the simultaneous decrease in the concentration of lignin and its solvent, initiating self-assembly of particle formation. During formation, the particles are assembled into spheres to minimize the surface area in contact with the nonsolvent phase.^61^ Acid precipitation resulted in systems with the highest dispersion degree, while solvent exchange generally yielded the lowest dispersion degree in the region where NP formation could be inferred based on size determination. All selected methods successfully produced particles with a substantial negative charge ranging from approximately −25 to −45 mV, indicating stable colloidal dispersion.^62^ Notably, solvent exchange with involvement of dialysis with a molecular weight cutoff (MWCO) of 14,000 Da generally achieved the highest negative charge dispersions, signifying favorable conditions for stable particle formation. This could arise from gradual self-assembly process^34^ enabling the organization of the negatively charged hydrophilic groups toward the particle’s surface, while the hydrophobic polymer segments preferably point inward to the core.

**Figure 2:**
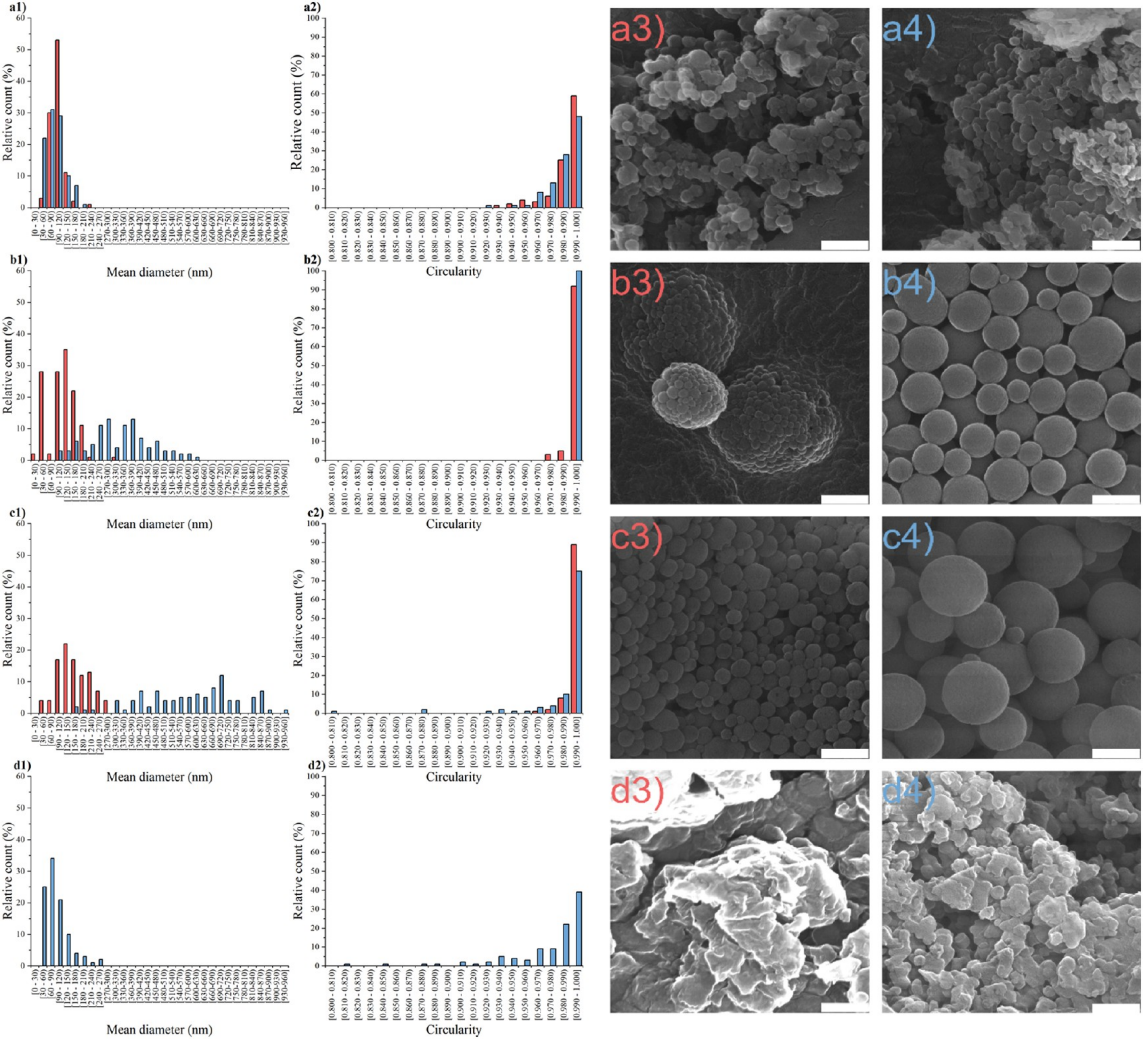
Influence of input lignin concentrations 0.1 mg mL^–1^ (red filled rectangle), 1.0 mg mL^–1^ (blue filled rectangle) on LigNPs (1) size, (2) circularity, and (3–4) morphology prepared using three distinct methods: (a) antisolvent precipitation, (b) dialysis solvent exchange 14,000 MWCO, (c) dialysis solvent exchange 3,500 MWCO, and (d) acid precipitation. Scale bars represent 500 nm.

Following the screening DLS measurements, we conducted scanning electron microscopy (SEM) and image analysis of lignin particles prepared using various methods at input concentrations of 0.1 and 1.0 mg mL^–1^. These samples were chosen based on dispersed phase hydrodynamic diameter determination, which indicates the presence of LigNPs (Figure 1). It should be noted that the preparation of LigNPs from low input concentrations is a limiting factor to scale up the process to an industrial level.^63^ As depicted in Figure 2, the sample prepared at an input lignin concentration of 1 mg mL^–1^ using acid precipitation contains LigNPs. The same was not confirmed for an input lignin concentration of 0.1 mg mL^–1^, which contained particles with irregular morphologies. Therefore, an image analysis was not performed for this sample. The resulting morphology, which resembles clusters composed of smaller particles, was also observed in other studies using the acid precipitation technique for preparation.^35,64,65^ In contrast, both samples from antisolvent precipitation exhibited similar size and morphology (Figure 2a). This is consistent with the hydrodynamic size determination (Figure 1) and suggests that within this concentration range the input concentration does not markedly affect the LigNPs’ morphology. The samples prepared via antisolvent precipitation and acid precipitation featured particles with diverse morphologies and indistinct boundaries between individual particles. Nevertheless, these were nanosized particles with median sizes of 99, 88, and 82 nm for antisolvent precipitation with input lignin concentrations of 0.1 mg mL^–1^, 1.0 mg mL^–1^ and acid precipitation at 1.0 mg mL^–1^, respectively. The unclear boundaries between nanoparticles may imply larger structures composed of nanoscale particles, as indicated by the behavior of these samples. Samples from antisolvent and acid precipitation partially sedimented within a short storage period, while dialysis-prepared samples formed a stable colloidal dispersion that partially sedimented only after prolonged storage at 4 °C, and the colloidal suspension was easily restored after sample homogenization.

**Figure 3:**
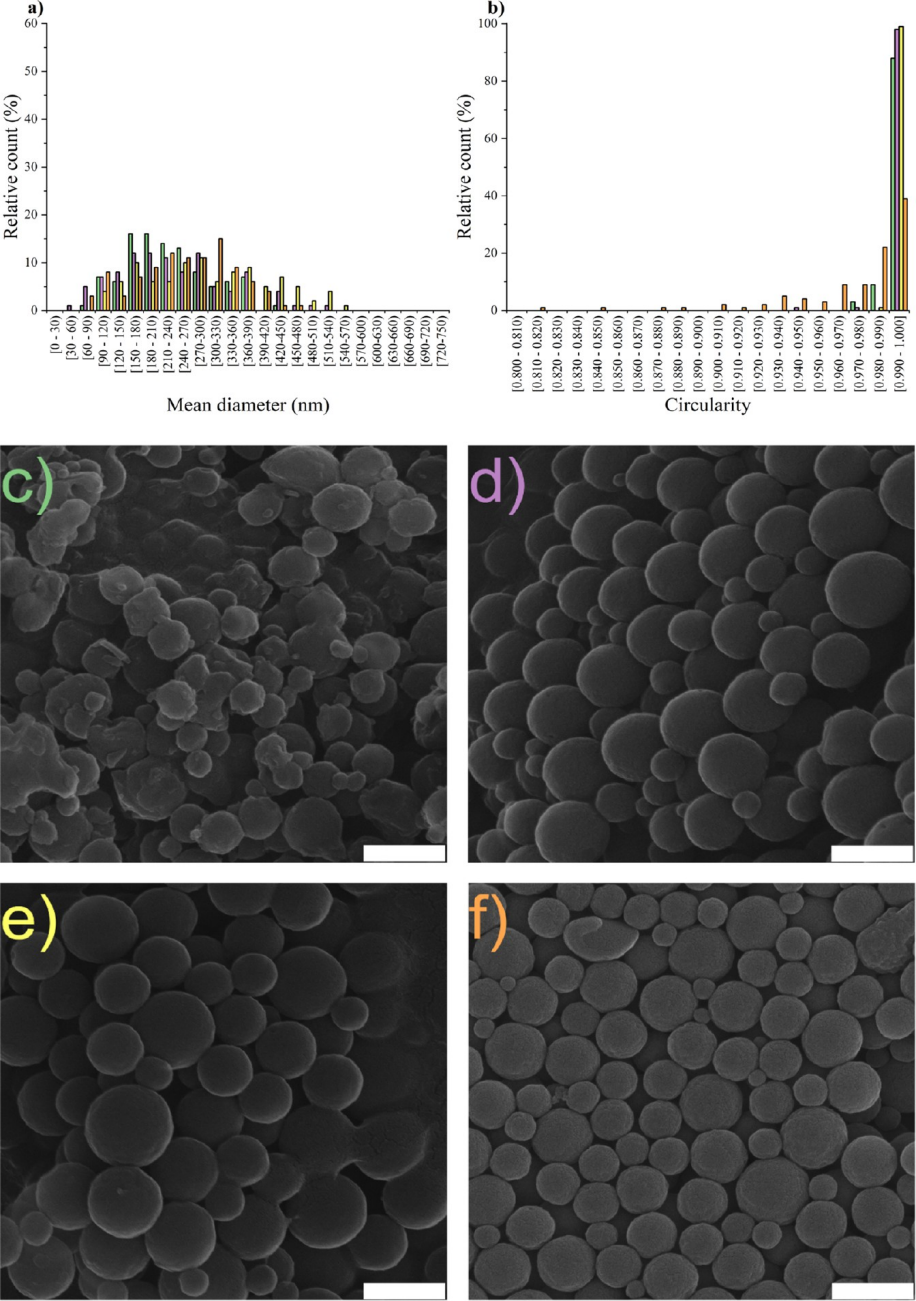
Influence of input lignin concentrations on LigNPs: (a) size, (b) circularity, and (c–f) morphology prepared using dialysis solvent exchange 0.25 mg mL^–1^, 14,000 MWCO (green filled rectangle), 0.50 mg mL^–1^, 14,000 MWCO (purple filled rectangle), 0.75 mg mL^–1^, 14,000 MWCO (yellow filled rectangle), and scale-up 0.50 mg mL^–1^, 10,000 MWCO (orange filled rectangle). Scale bars represent 500 nm.

Image analysis of solvent exchange samples confirmed a significant influence of input lignin concentration on particle size (Figure 2b1,c1).^66−69^ Particles prepared by solvent exchange exhibited regular spherical structures with well-defined boundaries, as evidenced by circularity determination, where over 75% of particles within solvent exchange prepared samples showed circularity greater than 0.990. Image analysis further revealed that the pore size of the dialysis membrane could be an additional parameter, along with the input lignin concentration, to control the size of LigNPs by solvent exchange. Generally, lower input lignin concentration and larger pore size of the dialysis membrane allowed the preparation of smaller particles as evident from median size determination −78, 339, 159, and 641 nm were determined for solvent exchange with 0.1 mg mL^–1^ 14,000 MWCO, solvent exchange 1.0 mg mL^–1^ 14,000 MWCO, solvent exchange 0.1 mg mL^–1^ 3,500 MWCO, and solvent exchange 1.0 mg mL^–1^ 3,500 MWCO, respectively. Given this influence, we focus on preparing the carrier of hybrid nanoparticles using solvent exchange with 14,000 MWCO, producing nanoparticles with input lignin concentrations of 0.25, 0.5, and 0.75 mg mL^–1^. Even within this relatively narrow concentration range, we demonstrated a dependence of particle size on the input concentration (Figure 3a–e), while maintaining a circular morphology. The median sizes for input concentrations of 0.25, 0.50, and 0.75 mg mL^–1^ were determined to be 213, 228, and 292 nm, respectively.

#### Choice and Characterization of LigNPs for Lignin–Silver Hybrid Nanoparticle Preparation

For the LigNPs designated for silver modification, we opted to use a dialysis solvent exchange with an input lignin concentration of 0.5 mg mL^–1^. This input concentration yielded LigNPs with the desired uniform morphology and the smallest median size of the particles (Figure 3). To meet the sample quantity requirement for subsequent experiments, we scaled up using commercially available dialysis cassettes with 10,000 MWCO (Figure 3f), avoiding a variance from the established mean molar mass of the lignin (*M*_w_ = 2.7 kDa, *M*_n_ = 1.4 kDa) used. Under these conditions, the median size increased to 256 nm compared to 228 nm for the same input concentration when using a dialysis membrane with a 14,000 MWCO. This increase results from the shrinkage of the membrane pores, which is consistent with previous findings (Figure 2). Simultaneously, there was a slight broadening of the circularity distribution during the scale-up phase compared to that of other LigNP samples prepared by dialysis with an input lignin solution volume of 10 mL (Figure 3b). The conversion yield of lignin to LigNPs using scaled-up dialysis solvent exchange with an input lignin concentration of 0.5 mg mL^–1^ was 52 ± 3%.

**Figure 4:**
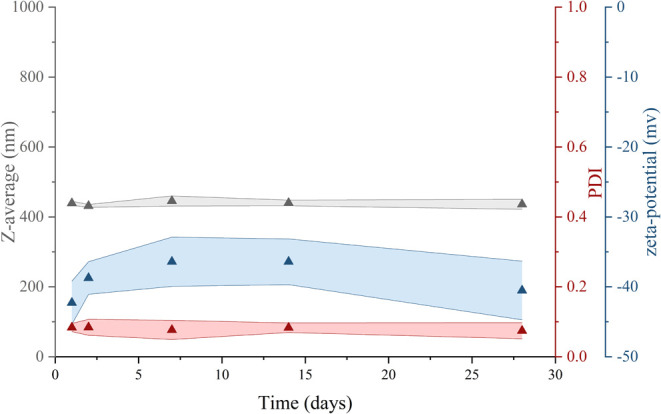
Time-dependence of the LigNP intensity-weighted mean hydrodynamic size (gray filled triangle), polydispersity index (PDI) (red filled triangle), and zeta-potential values (blue filled triangle). Colored areas represent the standard deviation values of three independent repetitions.

To introduce LigNPs into biomedical applications, it is important to maintain their long-term colloidal stability. The stability was investigated over 1 month of storage at 4 °C, during which no significant differences in the monitored characteristics of the colloidal dispersion were observed (Figure 4).

**Figure 5:**
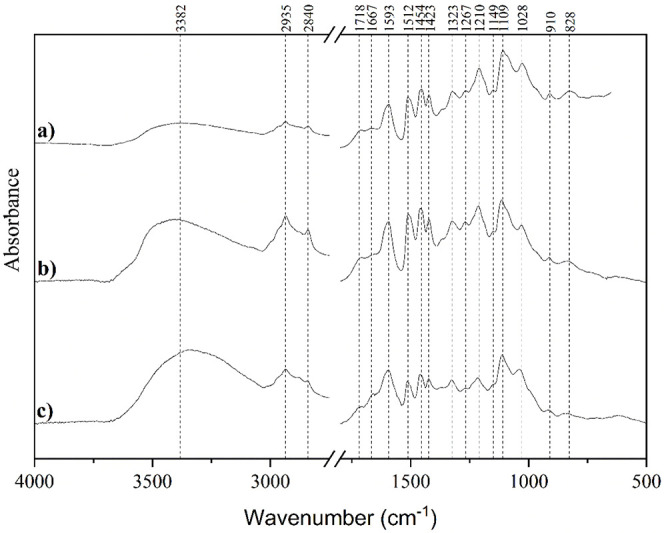
FTIR spectra of (a) raw lignin*, (b) LigNPs prepared using 0.5 mg mL^–1^ input lignin concentration and dialysis solvent exchange method 10,000 MWCO, and (c) supernatant after LigNPs separation. *The raw lignin spectrum is reprinted from Maršík et al.,^44^ under the Creative Commons Attribution 4.0 International.

In order to evaluate the lignin structure within the NPs, the samples were characterized by using FTIR spectroscopy. The lignin used in this study was the same as in our previous work on the synthesis of AgNPs.^44^ Analysis revealed identical functional groups represented by individual absorption maxima in both the LigNPs and the supernatant compared to the raw material (Figure 5). However, there are certain variations between the spectra of individual samples. Notably, a significant increase in absorption bands was observed within the range of 3700–3000 cm^–1^, indicative of stretching vibrations of alcohol and phenol −OH groups involved in hydrogen bonding.^35^ This increase in absorbance aligns with the proposed mechanism of LigNPs’ formation via the solvent exchange method, where large lignin fragments precipitate first and form nuclei. The process is followed by the growth of particles with hydrophilic moieties orienting toward the surface and small polar lignin fragments that adsorb onto the formed particles.^34^ Consequently, a decrease in the O–H stretching frequency with a broadening and intensification of the band occurs.^70^ As shown in the νOH band of Kraft lignin deconvolution in a study performed by Kubo et al.,^71^ we can hypothesize that the shift of absorption maxima of raw material from 3382 to 3402 cm^–1^ in the LigNP sample and 3348 cm^–1^ in the supernatant sample is a result of variation in hydrogen bonding or free hydroxyl groups within the complex lignin structure. The intensified peak in the supernatant sample compared to the rest of the spectra may result from the gradual decrease in lignin solubility with a decreasing concentration of acetone during nanoparticle formation. This transition is marked by a shift from long hydrophobic chains with a lower content of polar/hydroxyl groups to shorter polar chains.^34,61^ After the self-assembly formation is complete, the unincorporated lignin is separated during the isolation of the LigNPs (Figure S1). Indeed, it seems that hydrophobic moieties of polymer in the supernatant sample, represented by 2935, 2840, and 1454 cm^–1^ of non-cyclic (CH_2_)_*n*_ chains,^70^ are decreased compared to the LigNPs sample. Similarly, the bands at 1512, 1423, and 910 cm^–1^, attributed to aromatic skeletal ring vibrations,^72,73^ are also reduced. This indicates the presence of an unincorporated polar fraction.

Other peaks at 1718 and 1667 cm^–1^ represent nonconjugated and conjugated carbonyl stretching vibrations, respectively.^70,73−75^ Additionally, the 1323 cm^–1^ band is assigned to the C–O stretching from the syringyl monomer, characteristic of hardwood lignin,^71,72,76^ along with the peaks at 1109 and 828 cm^–1^ representing the H deformation and C–H bending of S units.^73^ The significant band in the region around 1210 cm^–1^ is associated in the literature with the absorbance of the guaiacyl band and is more intense in the case of organosolv lignin compared to Kraft.^77^ However, Ibrahim et al.^73^ attribute the peak in this region in the spectrum of beechwood lignin isolated by the organosolv process to OH in-plane bending in carbohydrates resulting from residual saccharides.^78^ This could also explain the broadening of the peak in the region of 1030 cm^–1^ in the supernatant sample compared with the LigNPs. In the case of lignin, the peak in this region is attributed to C–H deformation in guaiacyl and C–O deformation in primary alcohol.^35,71,76^ However, in the spectrum of cellulose and hemicellulose, a peak originating from C6–O6H stretching can be observed at the same position and dominates the entire spectrum.^78−80^ The presence of a hydroxyl-rich structure could provide an additional explanation for the previously discussed shift of the absorption band in the region 3700–3000 cm^–1^. The presence of polysaccharide (cellulose, hemicellulose) residues can significantly influence hydrogen bonding, potentially causing a shift in the maximum absorption to lower wavenumbers.^81^ In our isolation process, the sugar residues may be preferentially washed from the raw material into the supernatant along with other unincorporated lignin fractions due to their polar character. The remaining low-intensity peaks with assigned maxima of 1267 and 1149 cm^–1^ are attributed to the C–O and C–H stretching in the G-ring, respectively.^73,82^

### Fabrication of AgLigNPs

**Figure 6:**
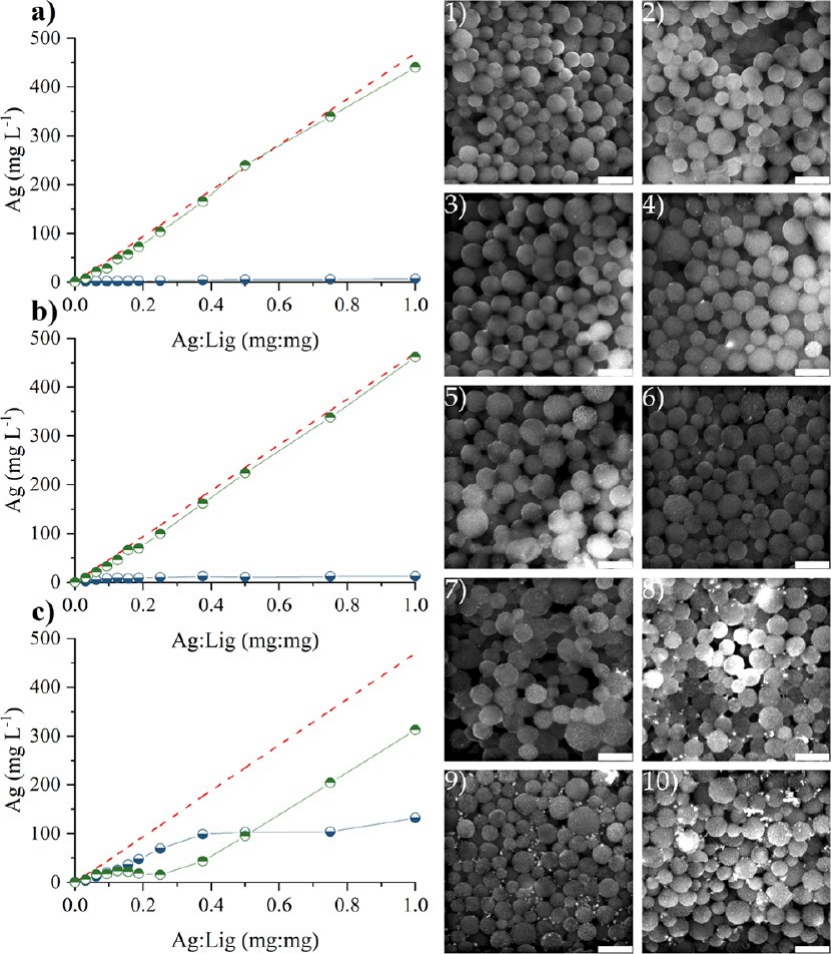
Influence of increasing pH on AgLigNPs’ preparation while maintaining constant concentration of LigNPs and Ag^+^ (1) control without NaOH addition, pH 6.2; (2) control without silver precursor (AgNO_3_) addition, pH 10.6; (3) pH 6.5; (4) pH 9.0; (5) pH 10.0; (6) pH 10.5; (7) pH 10.6; (8) pH 10.8; (9) pH 10.9; (10) pH 11.0 and influence of increasing Ag^+^ on AgLigNPs’ preparation (amount of silver entrapped on LigNPs (blue lower half filled circle) and in the supernatant (green upper half filled circle)) while maintaining constant concentration of NaOH (pH) and LigNPs (a) control without NaOH addition, pH 6.2; (b) pH 10.0; (c) pH 11.0. The red dashed line represents the calculated maximum mass concentration of Ag^+^. Scale bars represent 500 nm.

In the previous section, we introduced a method for preparing LigNPs. Solvent exchange dialysis resulted in a stable colloidal dispersion of LigNPs with a low polydispersity index and a highly negative charge at neutral pH, originating from the functional hydrophilic groups oriented toward the surface. The mean diameter of the LigNPs was determined to be 256 nm, with a circularity of 0.985. The regular spherical morphology of LigNPs provides a consistent and uniform surface, which is advantageous for maximizing the efficiency and uniformity of silver modification. Additionally, this is supported by the surface wrinkling of LigNPs observed via SEM (Figure 3f), providing a suitable surface for potential silver modification. This characteristic of LigNPs prepared using dialysis is in agreement with Kim et al.^83^ and is probably a consequence of the additional stacking of lignin residues during gradual solvent displacement. However, hydrophilic moieties, including hydroxyl groups oriented toward the surface of LigNPs in water as supported by FTIR (Figure 5), were ineffective in the silver precursor reduction under neutral pH and room conditions (Figure 6). This finding aligns with studies on the preparation of AgNPs using dissolved lignin as a reducing and capping agent, where formation is induced by an increase in reaction temperature^84^ or by elevating pH through the addition of a base.^85^ In our previous study on the preparation of AgNPs using dissolved lignin as a reducing and capping agent, the reduction of the precursor was facilitated by the addition of a base.^44^ Therefore, we decided to induce AgLigNPs synthesis by NaOH addition.

To mitigate potential dissolution or morphological alteration of the LigNPs in alkaline conditions, the reaction was performed under various pH levels adjusted by the addition of NaOH. As depicted in Figure 6, the increase of NaOH in the reaction mixture was positively correlated with the deposition of silver on the LigNPs’ surface while preserving the morphology. The rapid increase visually observed in the amount of trapped silver from pH 10.5 suggests that our synthesis likely proceeds through the autocatalytic reduction of Ag^+^ via Ag_2_O intermediates,^86^ which is stable at pH 10.5.^87^ Consequently, we focused on the silver content in AgLigNPs expressed as the mass ratio between the entrapped silver and the lignin in LigNPs (m_Ag_:m_Lig_). AAS analysis indicated a proportional rise in silver on the LigNPs at pH 11 up to an m_Ag_:m_Lig_ ratio of 0.25, beyond which a notable increase in silver concentration was observed in the supernatant. The yellow hue of the supernatant suggested the presence of AgNPs. Conversely, without the addition of NaOH and at pH 10.0, there was no significant entrapment of Ag on LigNPs (Figure 6a,b).

Overall, we introduced a suitable method for the preparation of AgLigNPs, achieving strong modification of the surface of LigNPs with silver. The reaction between silver and LigNPs does not occur spontaneously and needs to be induced, in our case, by the addition of a base. The effective modification of LigNPs by silver is pH-dependent, requiring a pH greater than 10.5. Simultaneously, up to pH 11, our LigNPs retained their morphology. Under these conditions, we detected a proportional increase of silver on the LigNPs’ surface up to an m_Ag_:m_Lig_ ratio of 0.25. The silver entrapment in the isolated sample represents a ratio of 0.12 ± 0.01 m_Ag_:m_Lig_, with 54 ± 5% of the silver from the precursor being entrapped on the surface of LigNPs. The prepared AgLigNPs were then subjected to further characterization, with respect to their silver component.

### Characterization of the Silver Component within AgLigNPs

**Figure 7:**
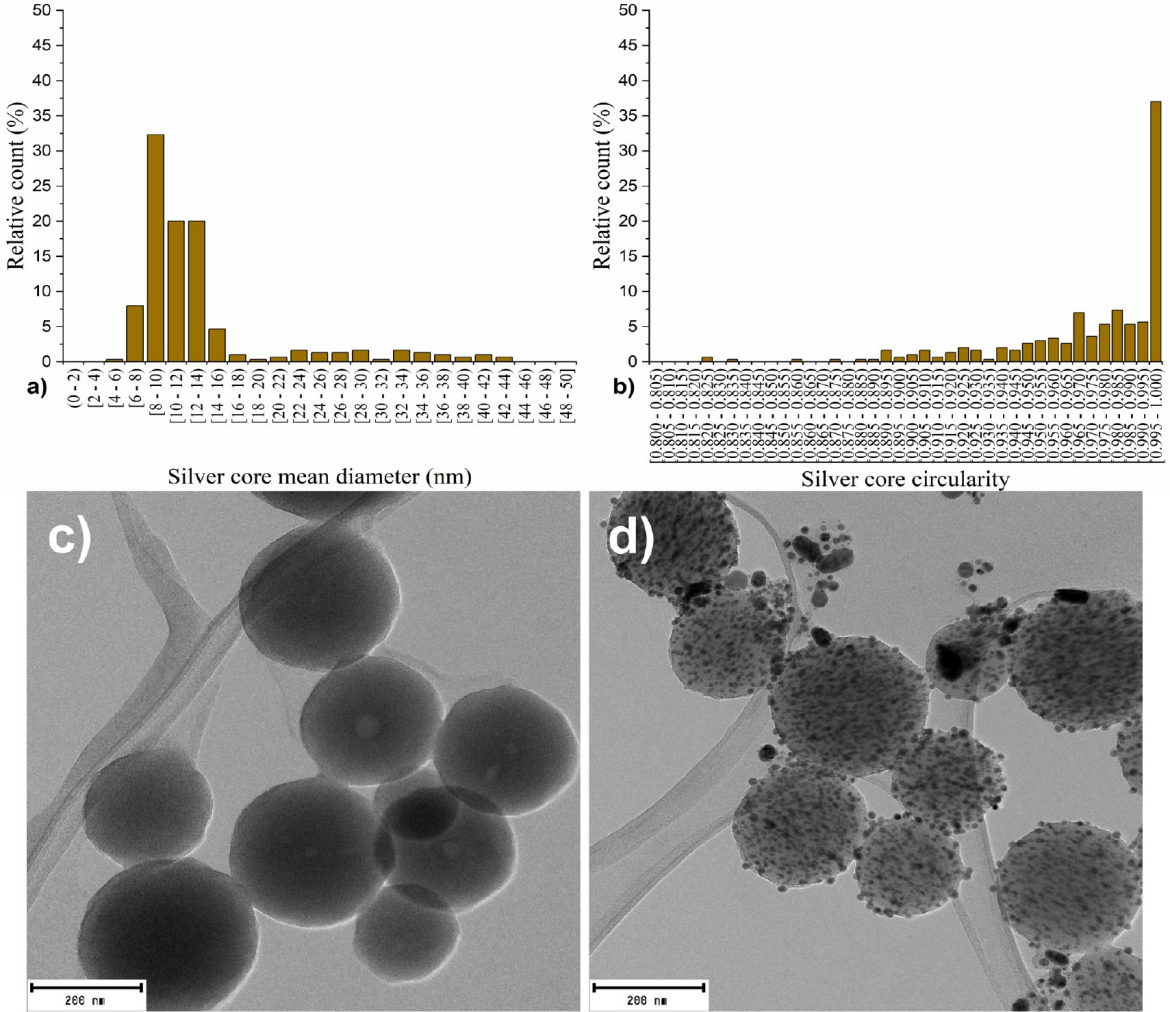
Characterization of silver component and morphology of AgLigNPs: (a) size distribution and (b) circularity of AgNPs. TEM images of (c) unmodified LigNPs and (d) AgLigNPs. Scale bars represent 200 nm.

For subsequent biological activity studies, it was necessary to characterize the silver component within the AgLigNPs. This is essential for the comparison with AgNPs prepared and characterized in our previous study.^44^ The preparation of AgNPs was based on the same principle as AgLigNPs, but the silver precursor was reduced and capped by a lignin alkaline solution instead of LigNPs’ dispersion. TEM microscopy confirmed the presence of captured silver in the form of AgNPs on the surface of the LigNPs (Figure 7). Size distribution analysis of the AgNPs cores revealed a size range from 4 to 44 nm; however, 85% of the AgNPs cores were within the 6–16 nm size interval. The median size of the silver core was 11 ± 8 nm, with a median circularity of 0.978 ± 0.035 (Figure 7). The cores are formed of a pure face-centered cubic structure of metallic silver (Figure S2), with a crystallite size of approximately 5 nm, as determined by the Scherrer equation. The size differences suggest that the sample is polycrystalline in nature.^88^

This characterization indicates that we have prepared two nanosystems containing AgNPs, whose silver components share key properties for further biological activity evaluation of the AgLigNPs.^44^ First of all, both samples are formed by pure metallic silver. Despite the wider size distribution of AgNPs on the surface of LigNPs, the determined median values are similar, i.e., 11 ± 8 nm for AgLigNPs and 12 ± 2 nm for unattached AgNPs. Also, crystallite size indicates a globally similar size, consistently 1 nm larger for AgNPs. The polycrystalline structure and high degree of circularity, which was determined to be 0.978 ± 0.035 for AgLigNPs and 0.985 ± 0.018 for unattached AgNPs, indicate that AgNPs in both nanosystems form faceted near-spherical shapes, which, under our preparation conditions, are energetically favorable morphologies.^89^

These comparisons of nanosystems within the silver component are essential because, as summarized by Menichetti et al. in their review,^90^ the effect of AgNPs is often evaluated in relation to the size, shape, and surface functionalization. The size of AgNPs, at a constant silver concentration, influences the number of particles available for interactions and affects cellular uptake. Additionally, smaller nanoparticles are generally more susceptible to the release of silver ions, which affects both the antimicrobial activity and cytotoxicity. Morphology influences direct contact with cell envelopes and may contribute to their mechanical damage. Finally, due to the use of the same lignin as the stabilizing agent, we do not expect alterations in the interactions of our NPs within the biological system originating from silver core functionalization. To summarize this, our nanosystems contain AgNPs with similar physicochemical properties, which will allow us to evaluate the benefit of the synthesis of AgNPs on the surface of LigNPs (AgLigNPs) compared to the dispersion of unattached AgNPs.

### Stability of Nanoparticles in Culture Media

As discussed above, our hybrid AgLigNPs, designed as an antimicrobial agent, retain the morphology of the LigNPs’ carrier after modification with silver under alkaline conditions. The silver component of AgLigNPs shares physicochemical properties with the dispersion of unattached AgNPs. To further reliably assess the biological activity of AgLigNPs, we conducted stability control experiments within the culture media by selecting three culture media. First, LB medium, commonly used for the cultivation of *P. aeruginosa*, the bacterial strain investigated herein.^46^ Second, TSB, a routinely utilized medium for evaluating antimicrobial effects against bacteria such as *Staphylococcus aureus*,^91−93^ the strain often tested in nanomaterial research due to its increasing spread of resistant strains.^94,95^ Lastly, DMEM medium enriched with 10% fetal bovine serum was employed to culture HaCaT and HEK cells. DMEM is commonly used for tissue cell cultivation and assessing the cytotoxicity of various nanomaterials.^96^ All experiments were performed under the same conditions used to evaluate the biological activity.

Stability controls are crucial for a better understanding the impact of NPs during *in vitro* antimicrobial and cytotoxicity testing. These tests often focus solely on material concentrations, neglecting the material–environment interactions.^13^ The influence of the culture media on AgNPs has been demonstrated by several studies.^13,97,98^ Regarding LigNPs, there is a limited understanding of their stability in culture media but their antimicrobial and cytotoxicity properties are currently being studied.^99^ Culture media can alter the characteristics of NPs, potentially inducing phenomena such as corona formation,^100,101^ aggregation, or dissolution, thereby releasing compounds which can increase toxicity to animal cells.^13,98^ We anticipate that the lignin used in preparing our nanosystems will provide enhanced stability compared with AgNPs synthesized via conventional physicochemical methods. Moreover, these experiments lay the foundation for understanding the consequence of attachment of AgNPs on the LigNPs, for which it is necessary to confirm the structural integrity of AgLigNPs under tested conditions.

#### Size and Charge

**Figure 8:**
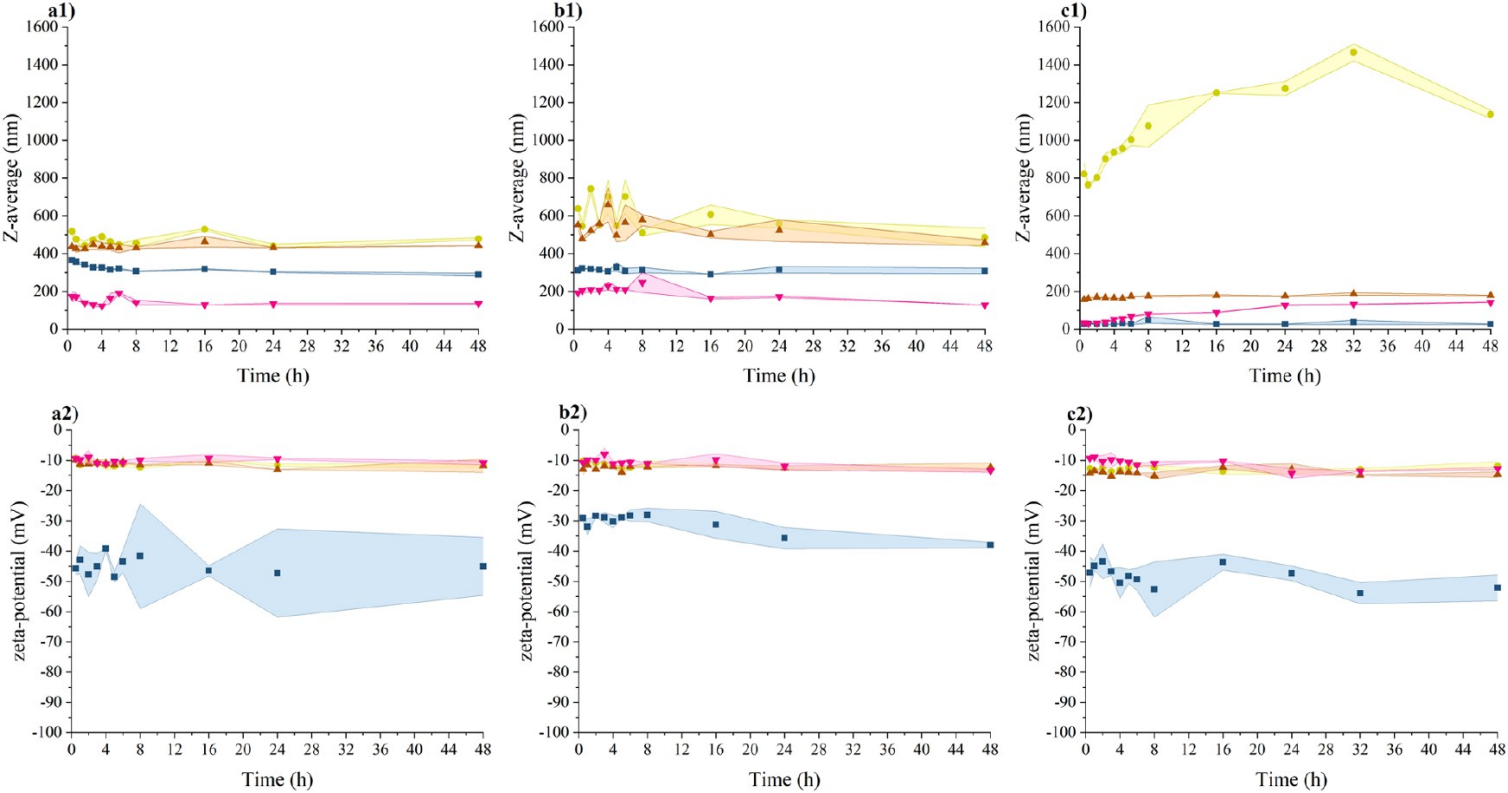
Time-dependence of (a) LigNPs, (b) AgLigNPs, and (c) AgNPs dispersions: (1) intensity-weighted mean hydrodynamic size and (2) zeta-potential values in LB (yellow filled circle), TSB (maroon filled triangle), DMEM + 10% FBS (pink down-pointing filled triangle), and UPW (blue filled square). Colored areas represent the standard deviation values of three independent repetitions.

From the determination of the hydrodynamic diameter, a general trend is observed across all three tested nanoparticles, with sizes increasing in the order of media from DMEM + 10% FBS, through TSB, to the LB medium. In almost all cases, equilibrium appears to be established within 30 min, and the determined hydrodynamic diameters were maintained for 48 h. An exception is noted with unimmobilized AgNPs in LB and DMEM + 10% FBS media, where a gradual increase in size is observed over time (Figure 8). This behavior aligns with previously reported findings, where AgNPs are known to form a protein corona,^102^ which increases the NPs’ size. As summarized by Pino et al.,^103^ corona formation depends on a number of factors, including size, charge, colloidal stability, protein type, temperature, incubation conditions, and time. In our experimental setup, the key differences are the size, colloidal stability, and type of protein present in the medium. The rest of the conditions were the same, including the total charge of the media dispersions, with the determined values consistently falling within a narrow range of −8 to −15 mV (Figure 8). The reduction in the value of the negative charge is caused by the constituents present in the media. The screened electrostatic repulsion of lignin promotes its affinity to proteins,^104^ which affects the electrostatic and steric stability of NP dispersions.^105−107^ The larger hydrodynamic diameters in LB and TSB media compared to water indicate that there is an interaction between NPs and proteins with a high probability of aggregate formation. The difference in the rate of reaching equilibrium between LigNPs, AgLigNPs, and AgNPs was caused by different colloidal dispersions’´ stability. The determined values of the hydrodynamic diameter in the DMEM + 10% FBS do not indicate aggregation, but the gradual increase in the hydrodynamic diameter of AgNPs suggests the formation of a corona.

**Figure 9:**
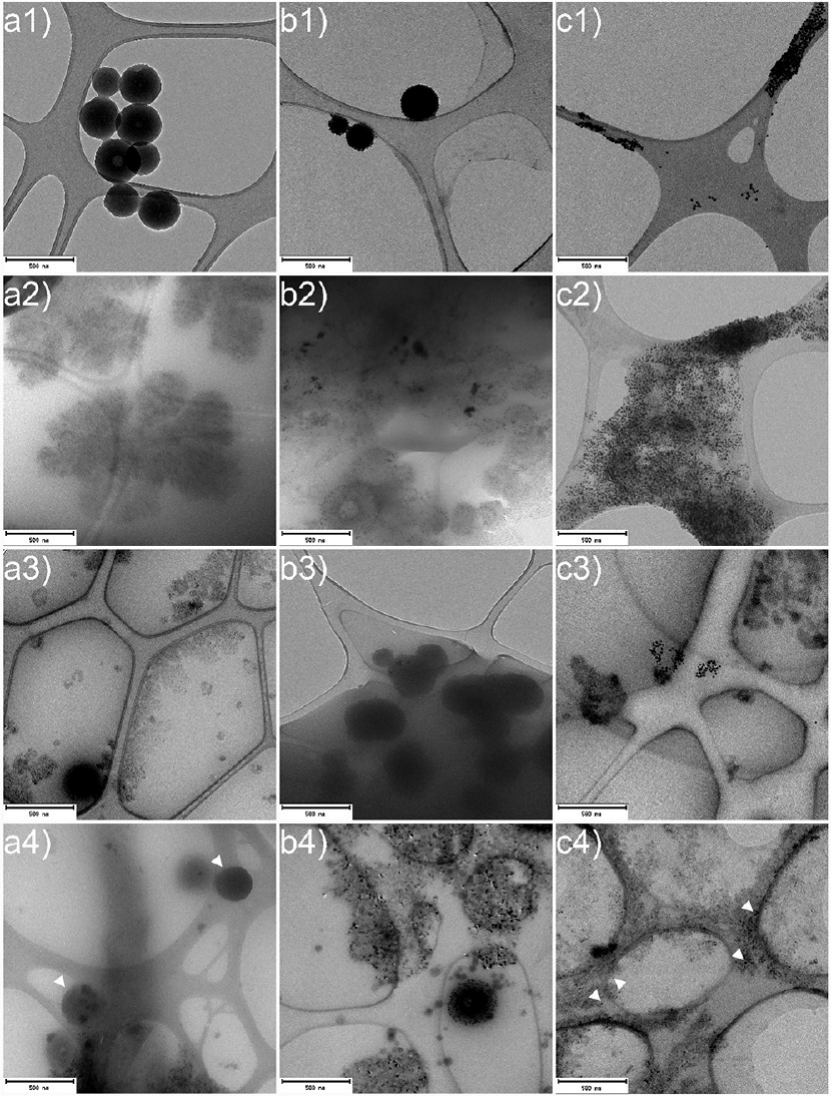
TEM imaging of (a) LigNPs, (b) AgLigNPs, and (c) AgNPs dispersions in (1) UPW, (2) LB, (3) TSB, and (4) DMEM + 10% FBS after 24 h of incubation. Scale bars represent 500 nm. White arrow heads indicate the location of NPs, which are difficult to distinguish from the components of the media.

As can be seen from Figure 9, upon transfer to TSB and LB medium while maintaining equal concentrations, a higher tendency to aggregate is evident, particularly in the LB medium, which aligns with the hydrodynamic diameter measurements (Figure 8). It is important to note that microscopy of diluted NPs in complex media affects figure quality, and without the presence of AgNPs on the surface, LigNPs could be hardly distinguished in LB medium (Figure 9). Moreover, the observation that AgLigNPs retain their surface modification by AgNPs even in such a complex environment is very encouraging. In the DMEM + 10% FBS medium, all tested NPs were randomly distributed without obvious mutual interactions. This can be observed from the representative TEM images (Figure 9a4,b4,c4), which suggest that at the set magnification that balanced visibility of all NPs and the surrounding area of the sample, no significant accumulation of NPs was detected, unlike in LB and TSB media. It should be noted that, following 24 h of incubation at 37 °C with constant agitation, we observed the release of minute silver nuclei from the surface of AgLigNPs (Figure 9b1), which are visible upon closer inspection of the surrounding area of the hybrid NPs. Additionally, it appears that the culture medium induces swelling of AgLigNPs, particularly noticeable in the LB medium (Figure 9b2). Otherwise, the integrity of NPs is preserved, and we did not observe their disintegration in culture media.

The surprising result in the hydrodynamic diameter of LigNPs and AgLigNPs is that the values are lower in DMEM + 10% FBS compared to those in water (Figure 8). Organosolv lignin, being a rather hydrophobic polymer due to its aromatic nature,^108^ aims to minimize unfavorable interactions between hydrophilic and hydrophobic blocks or between water and hydrophobic blocks.^109^ The noticeably higher determined hydrodynamic size compared to image analysis (256 nm) indicates that our nanoparticles form, despite the orientation of the hydroxyl groups on the nanoparticle surfaces, a partially stable dispersion in water. In other words, the electrostatic repulsion from the surface negative charge of (Ag)LigNPs (Figure 8) is outweighed by the hydrophobic–hydrophobic interactions, leading to an increase in size determined by DLS.^110^ This finding contrasts with our expectations, as a generally accepted rule suggests that nanoparticles with a charge greater than |30| mV should form a highly stable dispersion.^62^ However, in the presence of DMEM + 10% FBS media components, the hydrophobic interactions between the NPs weakened, causing them to disperse further apart, thereby reducing their size, as determined by DLS. This observation aligns with the microscopy findings (Figure 9), where all NPs incubated in water tended to cluster together on the microscopic grid. This is particularly evident in Figure 9a1, which shows the individual LigNPs closely associated with the polymeric lignin matrix appearing to bridge the gaps between them, creating a continuous, smooth transition from one NP to the next. Conversely, NPs in DMEM + 10% FBS were randomly distributed without evident interactions, as previously mentioned.

It still remains to be discussed the influence of the media composition (Table 1), particularly regarding the protein source affecting the hydrodynamic diameter within various media dispersions. Lignin is known for its irreversible interactions with proteins.^111^ The efficiency of lignin–protein adsorption varies with the type of protein and likely involves a combination of noncovalent interactions.^112^ Previous studies have demonstrated sorption of proteins such as BSA^113^ or soy proteins^114^ by lignin. Notably, soy protein, which is an amino acid source of TSB, has shown strong adhesion to lignin via cation−π interactions, as demonstrated by Zheng et al.^115^ It is plausible that our nanoparticles interact via NH_3_^+^–π interactions with incompletely hydrolyzed peptide chains present in the protein sources of the media. This interaction could bring individual NPs closer together and cause them to aggregate. The composition of LB and TSB includes tryptone (a pancreatic digest of casein) as the primary amino acid source. LB additionally contains yeast extract powder, while TSB is derived from digested soybeans. Despite TSB being richer in amino nitrogen (Table 1), suggesting a higher protein content in the medium, a greater increase in hydrodynamic diameter was generally observed in LB medium (Figure 8). Several factors contribute to this phenomenon. The higher ionic strength of LB may facilitate lignin–peptide interactions^104,112,114^ (Table 1). The yeast extract powder, a specific component of LB, may contribute to NPs’ aggregation. The amino acids phenylalanine, histidine, proline, and serine, identified as contributing to the peptide binding to lignin,^112,116^ are well represented in the yeast extract.^117,118^ However, the specific contribution of the amino acid composition cannot be confirmed with certainty. This uncertainty arises from the complex interactions influenced by the origin of lignin,^116^ the presence of other components affecting noncovalent interactions responsible for lignin binding,^112^ the process of protein hydrolysis as a source of amino acids in the culture medium,^119^ and the protein source itself. For example, in the case of soy protein, the resulting composition is highly variable and depends on the origin of the raw material.^120,121^ Moreover, yeast extract powder is generally rich in nucleic acids,^119,122^ serving as another potential source of NH_3_^+^–π interactions.

**Table 1:** Predominant Components of the Culture Media Influence the Stability of the Tested NPs^a^

Culture medium	Amino nitrogen [g L^–1^]	Total salt [g L^–1^]	Glucose [g L^–1^]
LB	0.382	10.4	0.0
TSB	0.698	7.5	2.5
DMEM	0.233^A^	10.9	4.5
10% FBS	0.576^P^ + 0.005^A^	0.04	0.15

a The values correspond to the resulting concentration in the cultivation medium. The total amino nitrogen contribution from FBS was estimated using the Jones factor for animal protein, assuming a 16% nitrogen content in pure protein.^123−125^ The protein content was derived from a comprehensive analysis of commercially available FBS by Lee et al., ranging from 33 to 39 g L^–1^ in concentrated solutions.^126^ P represents the amino nitrogen content in proteins, calculated using the Jones factor, while A denotes the amino nitrogen content of free amino acids

Unlike LB and TSB, DMEM contains free amino acids that cannot facilitate NP interconnections through NH_3_^+^–π interactions or other lignin–protein noncovalent interactions. The source of protein that may interact with NPs is 10% FBS supplement^127^ (Table 1). Despite this, we did not observe aggregation in the supplemented medium (Figure 9). The remaining stability could be attributed to the presence of a high amount of free amino acids, which prevent potential protein interactions. Additionally, the preserved folded conformation of proteins (BSA), influenced solely by heating to 56 °C by the manufacturer,^128−131^ likely contributed to their stability and reduced tendency for interactions compared to non-native states. Non-native states typically exhibit increased accessibility for interactions with surrounding molecules due to exposure of hydrophobic and backbone moieties that are typically hidden within the interior of the native fold.^132^ In addition, FBS proteins such as BSA could already have bound other hydrophobic ligands under physiological conditions, specifically free fatty acids,^133^ which are also present in the serum.^126^ This is further supported by the observation of Leskinen et al.,^112^ who demonstrated that globular proteins, the most abundant in FBS,^126^ bind less readily to the surface of LigNPs compared to random coil proteins.

#### Absorbance and Ion Release

The stability of the NP dispersions was further monitored using UV–vis spectrophotometry. We focused solely on the characterization of unattached AgNPs. This was adopted due to a methodological principle, which evaluates the typical absorption band for silver nanoparticles in the range of 400–500 nm.^134^ The absorbance maximum of our unattached AgNPs is 410 nm (Figure S3). This band can reveal potential AgNP enlargement, dissolution leading to the release of Ag^+^ ions, aggregation, and subsequent destabilization.^135−137^ However, in the case of AgLigNPs, the absorption band related to AgNPs is affected due to the sharing of conduction electrons between neighboring particles.^136,137^ This is manifested by a red-shift to 440 nm and a weakening of the absorption band (Figure S3). The apparently higher absorbance value at the absorption maximum is caused by the presence of LigNPs, which partially overlap with the AgNP absorption spectrum.

**Figure 10:**
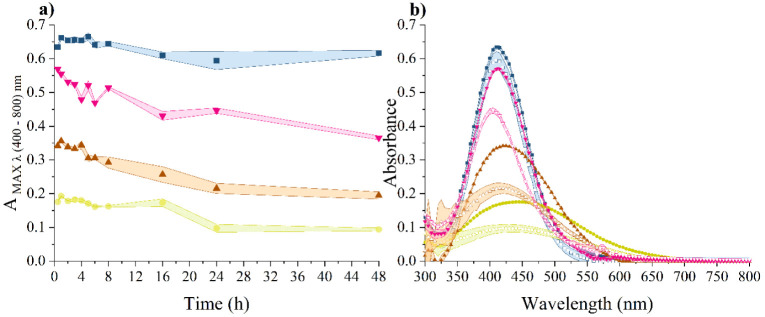
Time-dependence of AgNPs’ dispersion absorbance in LB (yellow filled circle), TSB (maroon filled triangle), DMEM + 10% FBS (pink down-pointing filled triangle), and UPW (blue filled square) plotted as (a) the maximum absorbance value ranging from 400 to 800 nm and (b) overall spectrum after 0.5 h of incubation (yellow filled circle; maroon filled triangle; pink down-pointing filled triangle; blue filled square) and after 24 h of incubation (yellow open circle; maroon open triangle; pink open down-pointing open triangle; blue open square) . Colored areas represent the standard deviation of three independent repetitions.

The commercial nanoparticle manufacturer considers a change in the intensity of the maximum absorbance exceeding 8% significant, warranting careful consideration of further applications of AgNPs based on specific needs.^138^ In our control sample of AgNPs dispersed in water, the absorption peak consistently appeared at 410 nm. After 24 h of incubation, there was a decrease in the absorption peak by 6.2 ± 4.2% (Figure 10). This is attributed to partial sorption of NPs onto the plastic surface^137,139^ mimicking the *in vitro* conditions of the biological activity testing. Overall, the dispersion of AgNPs is stable in water, as evidenced by no significant changes in absorbance, hydrodynamic size, zeta potential, PDI, or size determined by TEM after 1 month of storage at 4 °C without exposure to light (data not shown). However, changes in absorbance are observed upon transfer to complex culture media. When introduced to LB and TSB media, significant alterations in the absorption spectrum were noted, including intensity reduction, broadening, and a red shift of the maxima. Specifically, in LB media, the absorption maximum shifted over time to 435–445 nm, accompanied by a time-dependent decrease in absorbance of 44.4 ± 6.7% after 24 h (Figure 10). Similarly, in TSB media, the absorption maximum was consistently red-shifted at 420 nm with a decrease in absorbance of 43.0 ± 4.5% after 24 h. These spectral changes, characterized by broadening, red-shifting, and time-dependent decreases, indicate the gradual agglomeration of AgNPs. The effect of culture media is more pronounced in LB, which is consistent with observations from DLS and TEM (Figures 8 and 9).

**Figure 11:**
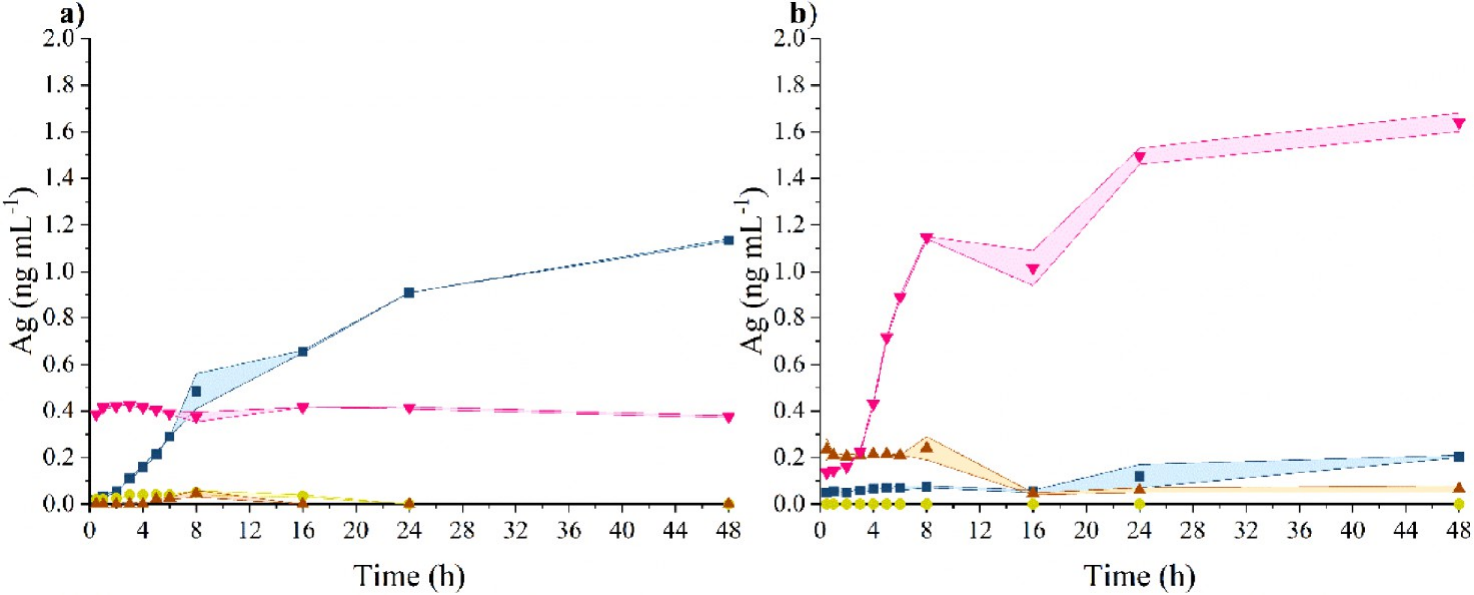
Time-dependence of (a) AgLigNPs and (b) AgNPs silver ion release in LB (yellow filled circle), TSB (maroon filled triangle), DMEM + 10% FBS (pink down-pointing filled triangle), and UPW (blue filled square) dispersions. Colored areas represent the standard deviation of two parallel dialysis runs.

In DMEM + 10% FBS, we also observed a time-dependent decrease in absorbance, amounting to a decrease of 23.1 ± 1.4% from 0.5 to 24 h of incubation. However, the absorption spectrum pattern in DMEM + 10% FBS closely resembles that in water, with a slight blue-shift of the maximum from 415 to 405 nm. The decrease in absorbance intensity can be attributed to a combination of partial sorption observed in the water and gradual interaction of media components with individual lignin-capped AgNPs. These interactions could affect Coulomb forces, leading to the alteration between nanoparticles and incident light during measurement,^140,141^ as supported by DLS and TEM data (Figures 8 and 9). Unlike in previous media, there is no indication of AgNPs’ aggregation in DMEM + 10% FBS, which supports our hypothesis regarding the influence of incompletely hydrolyzed proteins on NPs’ stability. Additionally, the slight blue-shift may suggest a reduction in the silver core diameter of AgNPs, due to the release of silver ions,^142^ as detected in the DMEM + 10% FBS medium (Figure 11b). Overall, 4.5 ± 0.1% of the total silver amount was released per 24 h. For the other media, no time-dependent release of ions was observed, and the amount released approximately corresponded to that observed during the incubation of AgNPs in water. The release observed in DMEM + 10% FBS could be attributed to the higher ionic strength of the medium,^143^ which contains the highest concentration of salts compared to LB and TSB (Table 1). Additionally, the release of silver ions from metallic silver in DMEM + 10% FBS can be supported by its higher concentration of glucose.^144^ Overall, it can be noted that there was a low release of silver ions from the unimmobilized AgNPs. For instance, Bouwmeester et al. observed a release of 17.4% from commercially available spherical nanoparticles with a size of 20 ± 2 nm in DMEM + 10% FBS after 24 h.^145^ This finding suggests that lignin serves as a suitable stabilizing and capping agent comparable to long-term stable phytosynthesized AgNPs prepared by Miškovská et al.^146^

In the case of hybrid AgLigNPs, consistent with free AgNPs, we observed an increased amount of released silver ions in DMEM + 10% FBS, while almost no release of silver ions was detected in the LB and TSB media (Figure 11). Interestingly, unlike unimmobilized AgNPs, no time-dependent release of silver was observed with hybrid AgLigNPs, and after 24 h, the released silver values were 1.2 ± 0.0%. Simultaneously, a gradual release was observed in UPW, with 2.7 ± 0.0% of the total silver released after 24 h of incubation. This gradual release in UPW may occur as colloidal silver ages toward equilibrium, a process that accelerates upon transfer to a fresh dispersant.^138,147^ Given the constituent values observed in DMEM + 10% FBS and the outcomes of previous experiments, we hypothesize that the presence of salts in DMEM + 10% FBS could induce the release of silver ions, followed by surface stabilization through the formation of AgCl_(s)_.^143^ In LB and TSB, release is prevented by the presence of partially hydrolyzed proteins, which promote aggregate formation. Lastly, the sudden release of silver observed in DMEM + 10% FBS is likely a methodological consequence,^148^ where the concentration gradient and thus the rate of dialysis are higher in DMEM + 10% FBS compared to UPW due to the presence of other components. Overall, the determined release of silver ions is generally low, and the AgNP cores attached to the LigNP surface remain stable in the complex environment of culture media.

### Antimicrobial Activity of Nanoparticles against *Pseudomonas aeruginosa* Planktonic and Adhering Cells

**Figure 12:**
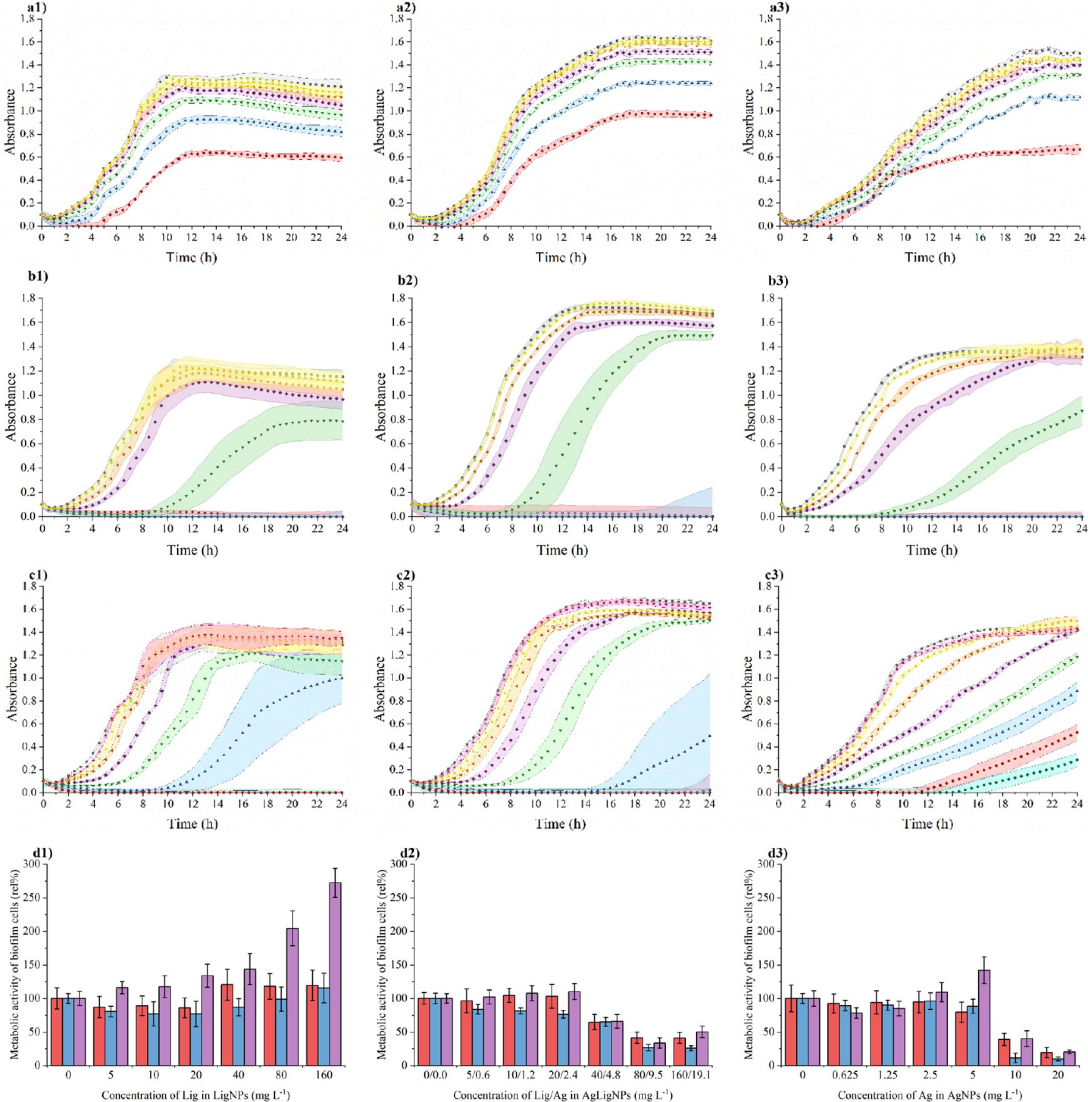
Effect of (a) LigNPs at a lignin concentration (mg L^–1^) 0 (gray filled square), 160 (red filled circle), 80 (blue filled triangle), 40 (green down-pointing filled triangle), 20 (purple filled diamond), 10 (brown left-pointing filled triangle), 5 (yellow right-pointing filled triangle), (b) AgLigNPs at a lignin/silver concentration (mg L^–1^) 0/0.0 (gray filled square), 160/19.1 (red filled circle), 80/9.5 (blue filled triangle), 40/4.8 (green down-pointing filled triangle), 20/2.4 (purple filled diamond), 10/1.2 (brown left-pointing filled triangle), 5/0.6 (yellow right-pointing filled triangle), (c) AgNPs at a silver concentration (mg L^–1^) 0.0 (gray filled square), 40.0 (green filled pentagon), 20.0 (red filled circle), 10.0 (blue filled triangle), 5.0 (green down-pointing filled triangle), 2.5 (purple filled diamond), 1.25 (brown left-pointing filled triangle), 0.625 (yellow right-pointing filled triangle), 0.3125 (pink filled star), on suspension growth of *P. aeruginosa* (1) ATCC 10145, (2) ATCC 15442, and (3) ATCC BAA-47 (PA01). Colored areas represent the standard deviation values of five parallel and three independent experiments. Effect of (d1) LigNPs, (d2) AgLigNPs, and (d3) AgNPs on adhering PA cells ATCC 10145 (red filled rectangle), ATCC 15442 (blue filled rectangle), and ATCC BAA-47 (PA01) (purple filled rectangle) in comparison to the untreated control. The presented values are the result of eight parallel and three independent repetitions.

To evaluate the benefit of the modification of LigNPs with AgNPs, we compared the growth kinetics of three different strains of *P. aeruginosa* exposed to unmodified LigNPs and unattached AgNPs, prepared and characterized in our previous work—see the part about Characterization of the Silver Component within AgLigNPs.^44^ The growth curves revealed that the minimum inhibitory concentration required to inhibit 50% of the bacterial population (MIC_50_) of LigNPs for the three tested strains was 160 mg L^–1^ (Figure 12), demonstrating high efficacy against suspension growth when compared to the MIC of 5,000 mg L^–1^ determined by Morena et al.^27^ for their prepared LigNPs against *P. aeruginosa* ATCC 10145. Nevertheless, we did not observe a minimum biofilm inhibitory concentration required to reduce biofilm development by 50% (MBIC_50_), and instead noted an increase in the metabolic activity of adhered cells, particularly in the ATCC BAA-47 strain (Figure 12). We attribute this increase to the oxidative stress generated by LigNPs, as reported in previous studies,^27,28^ which, according to Kim et al.,^149^ supports the overexpression of quorum sensing genes in planktonic bacteria, thereby promoting their transition to a biofilm growth mode.^150^

The modification of LigNPs with AgNPs increases the antimicrobial effect against both suspension growth and adhesion in the studied strains. For suspension growth, the MIC_50_ values of AgLigNPs shifted to <80/9.5 mg L^–1^, which was also the concentration required for complete inhibition (MIC) of PA suspension growth in all three tested strains. The minimum bactericidal concentration (MBC) of AgLigNPs was not determined within the tested range (up to 160/19.1 mg L^–1^). Regarding the effect on PA cell adhesion, we determined the MBIC_50_ to be 80/9.5 mg L^–1^ across all three tested strains. We assume that AgNPs on the LigNPs’ surface act against bacterial cells according to the generally proposed mechanism and primarily disrupt the bacterial envelopes.^151^ Furthermore, the presence of AgNPs addressed the increased adhesion observed with unmodified LigNPs (Figure 12). This finding is consistent with one of the antimicrobial effects of AgNPs, wherein exposure to AgNPs leads to the downregulation of quorum sensing genes, thereby suppressing biofilm formation.^31,152,153^

We further investigated the effects of modification by comparing AgLigNPs with those of unimmobilized AgNPs. The growth curves indicated a gradual lengthening of the lag phase with increasing silver concentration, reaching MIC_50_ at 10 mg L^–1^ for strain ATCC 15442 and 20 mg L^–1^ for strains ATCC 10145 and BAA-47 (Figure 12). Complete growth inhibition was observed at a silver concentration of 80 mg L^–1^ in the case of ATCC BAA-47 (data not included) and 20 mg L^–1^ for ATCC 15442 and ATCC 10145, with MBC values determined at 160 mg L^–1^ for strains ATCC 10145 and BAA-47, and 320 mg L^–1^ for strain ATCC 15442. MBIC_50_ values were consistently found to be 10 mg L^–1^ for all three tested strains (Figure 12).

Overall, it is evident that the same MIC_50_ values were achieved with approximately half the amount of silver used in AgLigNPs compared to unimmobilized AgNPs (Figure 12). When comparing MIC values, the amount of silver required was reduced by two- to eight-fold in AgLigNPs compared to AgNPs. This enhanced effect may be attributed to several factors, including the synergistic action of LigNPs with attached AgNPs (Figure 12). Given that nanoparticle antimicrobial effects are often discussed in terms of surface interactions between the nanoparticle and the cell, it is plausible that other mechanisms contribute to this enhancement. Based on the previous section about the characterization of the silver component within AgLigNPs, we can rule out the influence of the silver oxidation state (Figure S2) and the difference in surface area (Figure 7) as an explanation for the increased antimicrobial effect. Although the number of silver nanoparticles should be almost the same in both samples, the silver component in AgLigNPs is more polydisperse compared to unattached AgNPs, which, as indicated in previous work, may be the reason for the increased effect.^154^ These observations underline the morphology-dependent antimicrobial effects, as AgLigNPs retain their integrity in the culture medium (Figure 9) and the AgNPs act as part of LigNPs with a median size of 256 nm (Figure 3). Previous studies have observed damage to the membrane surface caused by AgNPs^154,155^ and LigNPs,^27^ supporting this hypothesis. However, we must point out that aggregation of both silver-containing nanosystems was observed in the LB medium after 24 h of incubation, complicating the reliable data evaluation in such a complex material–environment–cell system. Given that the aggregation of unattached AgNPs was time-dependent (Figures 8 and 10), it is reasonable to assume that unaggregated AgNPs interact simultaneously with media components and with cell-surface proteins. Furthermore, the release of silver ions from the nuclei stabilized by lignin was not observed in the LB medium. This observation suggests that the effect of our nanoparticles is either strongly morphology-dependent or acts via a Trojan horse mechanism,^156,157^ with partial silver ion release occurring upon contact with the bacterial membrane, where only submicromolar concentrations of Ag^+^ are needed for destabilization.^158^ Finally, the consistent Ag concentration in both nanosystems to achieve MBIC_50_ suggests that the effect against adhesion and biofilm development is strongly dependent on the concentration of Ag. In addition to the discussed effect of AgNPs on quorum sensing, the attachment of planktonic PA cells to surfaces is related to the growth phase and occurs during the exponential growth when cells are phenotypically heterogeneous.^159^ This correlates with our observation that the decrease in adhered cells is associated with the gradual lengthening of the lag phase in the Ag-containing nanosystems. This likely explains the slightly higher effect related to the silver concentration observed in the case of AgLigNPs compared to AgNPs, where a decrease in adhered cells was noted at a concentration of 40/4.8 lignin/silver mg L^–1^, whereas for AgNPs, this concentration was found at 10 mg L^–1^ of silver. In the case of unmodified LigNPs, we did not observe a significant prolongation of the lag phase, and the exposed PA cells could grow and adhere in a time similar to that of the control.

### Evaluation of Nanoparticles’ Cytotoxicity

We investigated the cytotoxic effects of various concentrations of prepared NPs on two distinct cell cultures. HEK-293, a cell line derived from embryonic kidney cells, was selected as a model to assess the overall cytotoxicity in human cells. Additionally, we used HaCaT, an immortalized human epidermal keratinocyte cell line due to its relevance for the potential application of AgLigNPs in the prevention and treatment of skin bacterial infections.

**Figure 13:**
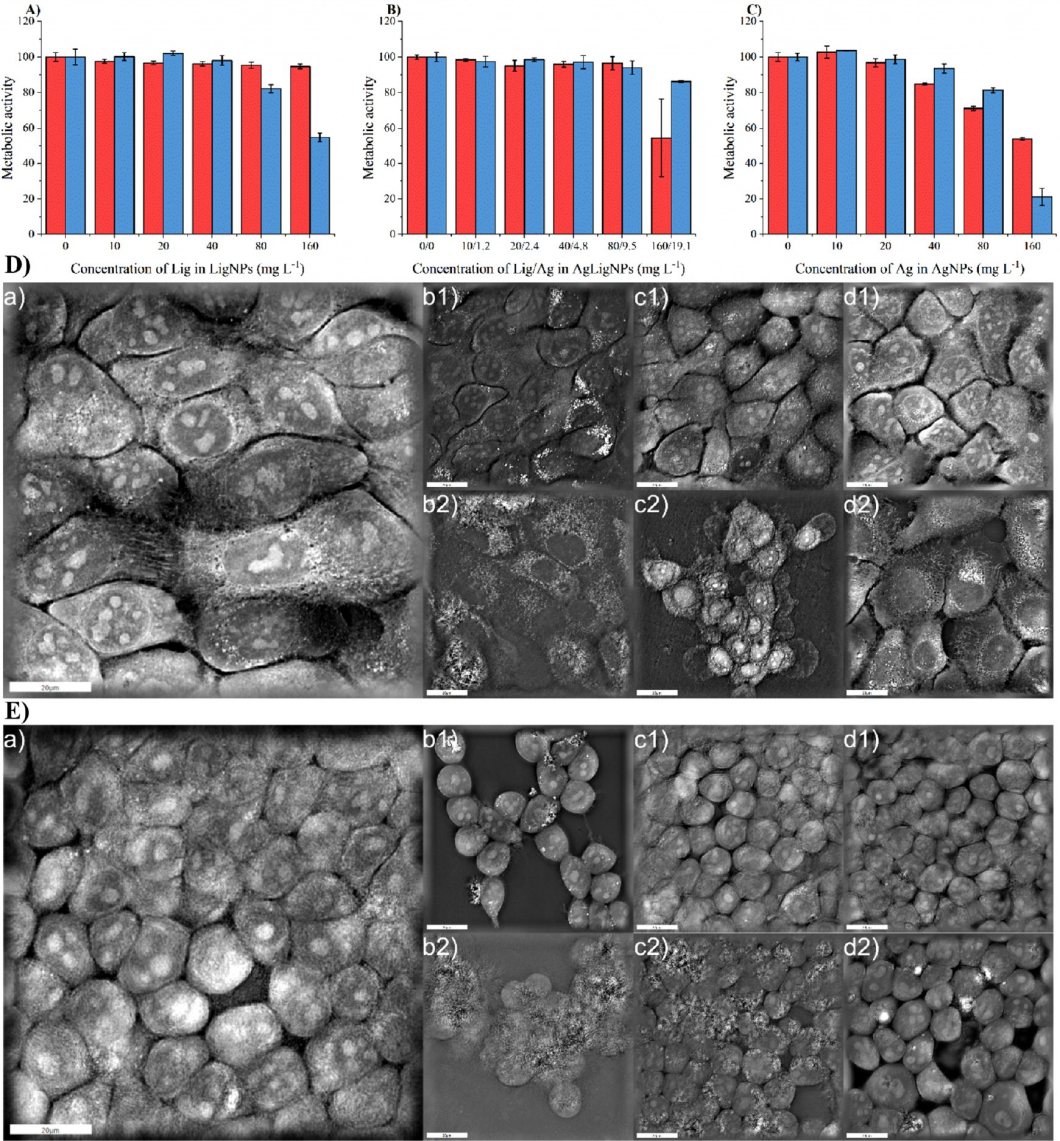
Effect of (A) LigNPs, (B) AgLigNPs, and (C) AgNPs on HaCaT (red rectangle) and HEK-293 (blue rectangle) cell cultures in comparison to the untreated control. The presented values are the result of three parallel experiments and one repetition. Morphology alterations of (D) HaCaT and (E) HEK-293 cell cultures after exposition to (b) LigNPs, (c) AgLigNPs, and (d) AgNPs at lignin/silver concentration (1) 20 mg L^–1^ and (2) 80 mg L^–1^ compared to the (a) untreated control.

Our results indicate that up to the concentration of 80 mg L^–1^ lignin in LigNPs, 80/10.8 mg L^–1^ lignin/silver in AgLigNPs, and 80 mg L^–1^ Ag in AgNPs, there was no significant toxicity observed (Figure 13). Visual observation also confirmed the typical morphologies of the cell cultures at a concentration of 20 mg L^–1^ of lignin in LigNPs, 20/2.4 mg L^–1^ of lignin/silver in AgLigNPs, and 20 mg L^–1^ of Ag in AgNPs. These concentrations have previously been shown to affect *P. aeruginosa* growth kinetics (Figure 12).

However, at a LigNP concentration of 160 mg L^–1^, we observed a nearly 50% decrease in metabolic activity in HaCaT culture, while the metabolic activity of HEK-293 cells was preserved. Microscopic analysis revealed strong adherence of LigNPs to cell membranes, impacting the image quality despite repeated rinsing (Figure 13). Nevertheless, cells in direct contact with LigNPs maintained their morphology. Given that lignin is generally regarded as nontoxic in NP form, with some studies suggesting safety at concentrations over 1,000 mg L^–1^,^27,160^ the observed reduced metabolic activity in HEK-293 cells may stem from slowed cellular processes and subsequent proliferation due to nanoparticle–membrane interactions. Furthermore, apoptotic bodies of HEK-293 cells were observed by microscopy, likely due to oxidative stress (Figure S4).^161^ Although lignin is generally considered an antioxidant, its phenolic nature can exhibit prooxidative effects depending on factors such as concentration, pH, and the presence of transition metals. The prooxidative behavior of phenolate ions can promote the formation of hydroxyl radicals,^162^ contributing to membrane lipid peroxidation.^163^ Similar prooxidative activity was also observed in zerovalent silver nanoparticles forming hydroxyl radicals.^164^ The interaction between lignin and silver nanoparticles in AgLigNPs may alter the overall cellular morphology, especially evident in HaCaT cells at 80/9.5 mg L^–1^ (Figure 13). Despite this, the metabolic activity of HEK-293 cells remained almost unaffected at the highest tested concentration, possibly due to reduced affinity or direct contact of LigNPs as a result of their Ag modification. This observation is supported by microscopy images showing HEK-293 cells in direct contact with adhered AgLigNPs on the cell surface, maintaining their morphology. Overall, our results further confirm that human cell lines can vary in their sensitivity to lignin-based NPs.^160^ We hypothesize that HEK-293 cells could be more sensitive to the presence of phenols on the surface of LigNPs, as these phenols participate in binding with AgNPs, thereby reducing direct contact with the polar heads of the cell membrane in AgLigNPs.

In the case of unattached AgNPs, a significant reduction in metabolic activity was observed at 160 mg L^–1^ silver in both cell cultures (Figure 13). Morphological changes were more pronounced in HaCaT cells, with evident damage to cell membranes and perinuclear regions. HEK-293 cells showed preservation of integrity but exhibited cellular enlargement. (Figure 13). Cellular enlargement can be a source of hypertrophy, which was also suggested to be caused by AgNPs by previous in vivo studies.^165,166^

## Conclusion

Based on the initial screening of three different LigNP preparation approaches, we identified conditions for preparing spherical LigNPs suitable for further modification by silver. We found the dialysis solvent exchange method advantageous for several reasons, which include the production of LigNPs with regular morphology, long-term stability of the aqueous dispersion, stability maintained in an alkaline environment, the orientation of reducing hydrophilic groups toward the surface to facilitate silver precursor reduction, and the wrinkled surface that provides sites for AgNPs’ attachment. Next, we determined the preparation conditions that resulted in robust LigNPs’ coverage by AgNPs, a crucial parameter to target bacterial envelopes. Furthermore, we demonstrated the benefit of hybrid AgLigNPs compared with control samples of blank LigNPs and unattached AgNPs prepared using the same lignin as a reducing and capping agent. Our results showed that hybrid AgLigNPs exhibited lower MIC values against PA compared with both the lignin content in blank LigNPs and the silver content in unattached AgNPs. This was supported by characterization of the AgNPs which revealed that the structure and size of both nanosystems are similar, and the enhanced antimicrobial effect results from the LigNPs modification. In addition, the conducted stability tests within culture media commonly used for antimicrobial testing revealed a tendency for all three nanosystems to aggregate, likely due to noncovalent interactions between partially hydrolyzed proteins and lignin. Despite this, the overall nanoparticle morphology was preserved, and the presence of lignin was beneficial in stabilizing the silver cores, as no significant release of silver ions was detected. Conversely, within a DMEM + 10% FBS used for cytotoxicity evaluation, the NPs remained stable and did not aggregate. This suggests that our NPs could retain their stability under in vivo conditions, which are mimicked by DMEM + 10% FBS.^167,168^ Finally, both silver-containing NPs demonstrated nontoxicity to HEK-293 and HaCaT cells at concentrations that completely inhibited suspension growth and reduced biofilm formation by 50%. This supports their potential for further valorization in the biomedical field.

## Experimental Section

### Lignin Isolation Process

Lignin was isolated via the organosolv process from beech sawdust (Lignocel HBS 150/500, Rettenmaier Sweden KB JRS, Helsingborg, Sweden) as previously described.^44^ In brief, sawdust was treated in a 60% v/v ethanol in water solution at 180 °C for 1 h with 1% w/w_dry biomass_ sulfuric acid at a liquid-to-solid ratio of 10 (mL g^–1^). The treatment took place in an air-heated multidigester (Haato, Vantaa, Finland), and after the treatment, the pretreated pulp (containing mainly the cellulose) was separated from the slurry via vacuum filtration. The filtrate was then processed in a rotary evaporator to remove the ethanol, rendering lignin insoluble in the aqueous solution, which was recovered via centrifugation at 10,000*g* for 15 min at 4 °C (5804R; Eppendorf, Hamburg, Germany), followed by freeze-drying (Lyoquest; Telstar, Terrassa, Spain). Lignin was then stored at room temperature until further use.

### Fabrication of LigNPs

For the LigNPs’ fabrication, three methods were employed: antisolvent precipitation, solvent exchange, and acid precipitation. In the antisolvent precipitation method, ultrapure water (UPW) was added to 10 mL of a 0–20 g L^–1^ lignin solution in 60% (v/v) acetone, with continuous stirring at 500 rpm, following the equation


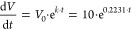


 is rate of change of volume over time (mL min^–1^), *V*_0_ is the initial volume at *t* = 0 (mL), *k* is the rate constant (min^–1^), *t* is the time (min), *V*_HCl_ is the amount of added acetic acid (mL), and *C*_Lig_ is the mass concentration of lignin (mg mL^–1^).

Water addition was stopped upon reaching a total volume of 60 mL. For solvent exchange, 10 mL of a lignin solution (0–20 g L^–1^) in 60% (v/v) acetone was transferred to a dialysis membrane with molecular weight cutoffs (MWCO) of 14,000 (Sigma-Aldrich, USA) or 3,500 Da (SnakeSkin, Thermo Fisher, USA). During the scale-up phase, a dialysis cassette with an MWCO of 10,000 Da (Slide-A-Lyzer G3, Thermo Fisher, USA) was employed, filled with 70 mL of a 0.5 g L^–1^ lignin solution in 60% (v/v) acetone. Dialysis was conducted against a volume of distilled water 300 times higher for 24 h with constant stirring at 300 rpm. Acid precipitation followed the method introduced by Frangville et al.^65^ To 10 mL of lignin solution in ethylene glycol with a concentration of 0–20 g L^–1^, 0.25 M HCl was added dropwise with continuous stirring at 500 rpm, following the equation *V*_HCl_ = 0.01*C*_Lig_. Afterward, the mixture was stirred for an additional 20 min, followed by dialysis using a membrane with a cutoff molecular weight of 3,500 Da (SnakeSkin, Thermo Fisher, USA) for 24 h under constant stirring at 300 rpm at 25 °C against 300 times the volume of distilled water. The prepared samples of the lignin particles were isolated before further characterization using three repeated centrifugations (30 min, 10,000*g*, 10 °C). The obtained pellets were resuspended in UPW.

### Fabrication of AgLigNPs

The synthesis of AgLigNPs involved a sequential reaction among LigNPs, AgNO_3_, and NaOH at various ratios. The reaction proceeded as follows: silver nitrate was first added to the LigNPs dispersion, followed by continuous stirring at 180 rpm at room temperature. Subsequently, a 0.035 M NaOH solution was added to the mixture. Initially, the effect of pH on AgLigNPs synthesis was investigated while maintaining a constant concentration of LigNPs (*C*_Lig_ = 520 ± 27 mg L^–1^; 1,000 μL) and AgNO_3_ (*C*_Ag_ = 56,100 mg L^–1^; 4.5 μL). The pH of the solution was determined using only the LigNPs’ dispersion and the specified amount of 0.035 M NaOH. To maintain the reaction volume, the solution was supplemented with UPW before the NaOH addition (total NaOH + UPW = 60 μL).

The appropriate amount of Ag for modification was determined in the total reaction volume of 5,350 μL, as the mass ratio between a constant amount of LigNPs (*C*_Lig_ = 520 ± 27 mg L^–1^; 5,000 μL) and varying amounts of Ag in AgNO_3_ (*C*_Ag_ = 56,100 mg L^–1^), while maintaining a constant amount of NaOH (*C*_NaOH_ = 0.035 M; 300 μL). To maintain the reaction volume, the solution was supplemented with UPW before NaOH addition (total AgNO_3_ + UPW = 50 μL).

Nanoparticles used for further characterization, antimicrobial efficacy determination, and cytotoxicity assessment were prepared at an m_Ag_:m_Lig_ ratio of 0.25 under pH 11. The synthesized AgLigNPs were always isolated for further characterization through six repeated centrifugations (30 min, 10,000*g*, 10 °C). The resulting pellets were resuspended in UPW and stored in the dark at 4 °C.

### Lignin and Silver Content Determination

The concentration of lignin in LigNPs was quantified following the protocol described by Figueiredo et al.^35^ A calibration curve was constructed using raw lignin dissolved in 60% (v/v) acetone across a concentration range of 0.1–1.0 mg mL^–1^. For analysis, LigNP samples in UPW were dissolved in pure acetone in a 2:3 ratio (v/v). Subsequently, 1.8 mL of UPW and 150 μL of Folin–Ciocalteu reagent were mixed with 50 μL of the prepared samples. After a 6 min incubation, 500 μL of a sodium carbonate solution (20% w/w) was added, followed by thorough mixing and a 30 min incubation at 40 °C. The absorbance of 200 μL aliquots was then measured at 760 nm.

The mass concentration of silver in AgLigNPs was determined by utilizing an Atomic Absorption Spectrometer (AAS) Agilent 280 FS AA (Agilent, USA). Initially, 200 μL of the sample of isolated AgLigNPs or the first supernatant collected during the isolation process was mixed with twice the volume of 4% HNO_3_ (v/v). The resulting solution was diluted with UPW to achieve a final volume of 10 mL, and those prepared solutions were analyzed.

### DLS Analysis and Zeta Potential Determination

The hydrodynamic diameter (Z-average), polydispersity index (PDI), and zeta potential were measured by using a Zetasizer Pro instrument (Malvern Panalytical, UK). Samples of LigNPs, AgLigNPs and AgNPs were diluted to a concentration of 10 mg L^–1^ lignin/lignin/silver content in the required medium. For the evaluation of NP media stability (LB, TSB, DMEM + 10% FBS, UPW), the diluted samples were incubated at 37 °C with continuous agitation at 150 rpm by using orbital shaking. At specific time points (0.5–48 h), the samples were loaded into folded capillary zeta cells DTS1070 (Malvern Panalytical, UK) and prior to measurement, the samples were allowed to equilibrate at 25 °C. Data were processed using ZS XPLORER software v.2.3.0.62 (Malvern Panalytical, UK).

### Morphology, Size Distribution, and Circularity Determination

To investigate the size distribution and morphology of LigNPs, the particles were visualized using a scanning electron microscope (SEM) MIRA 3 (FE-SEM, CZ) with a perpendicular in-beam secondary electron detector at high magnifications up to 100 kx and accelerating voltages of 30 kV (Schottky emitter). The NP samples for SEM measurement were prepared by drop-casting a dispersion of NPs onto the carbon tape, and the NP clusters were formed after evaporation of the dispersant at the droplet interface. NPs were also measured from the powder form obtained by lyophilization of the dispersion. To avoid unwanted charging, the LigNPs were coated with 5 nm of gold in a Denton Desk V HP TSC system, and AgLigNPs were characterized without additional coating.

To investigate the size distribution and morphology of attached AgNPs on LigNPs surface and the stability of LigNPs, AgLigNPs and LigNPs in culture media, the samples were visualized using an EFTEM Jeol 2200 FS transmition electron microscope (TEM) (JEOL, JP) operating at an electron beam energy of 200 kV. The NP samples for TEM measurement were prepared by drop-casting dispersion onto the copper grid, and the NP clusters were formed after evaporation of the dispersant at the droplet interface. For the evaluation of the LigNPs, AgLigNPs, and AgNPs stability within media (LB, TSB, DMEM + 10% FBS, UPW), the diluted samples to a concentration of 10 mg L^–1^ lignin/lignin/silver content were incubated for 24 h at 37 °C with continuous agitation at 150 rpm using orbital shaking.

The SEM and TEM images were manually analyzed using ImageJ 1.53e open-source software, running on Java 1.8.0_172. For each NP, the major 2*a* and minor 2*b* axes were measured, enabling calculation of the ellipse area *A* (eq 1). This computed area was then compared with the area obtained using the freehand drawing tool. The mean particle diameter was subsequently calculated as in eq 2. Circularity (*c*) was determined as the ratio of the particle area (*A*) to the area of a circle with an equivalent perimeter (*p*) (Equation 3).^169^ The particle perimeter (*p*) was approximated by using eq 4. The measurement protocol was performed always for 100 LigNPs prepared by distinct methods and 300 AgNPs on the AgLigNPs surface, each across three independent repetitions.
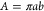
1
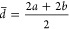
2
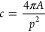
3
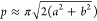
4

### LigNPs Characterization by FTIR

Fourier transform infrared (FTIR) spectra of lyophilized samples were obtained using a Nicolet 6700 FTIR spectrometer (Thermo Fisher, USA), equipped with a diamond ATR GladiATR (PIKE, USA) and a DTGS KBr detector. Spectral data were collected over the range of 4000–500 cm^–1^ with a resolution of 4 cm^–1^, using 64 scans and Happ-Genzel apodization. Baseline corrections were made by subtracting air humidity and CO_2_ signals from the spectra.

### AgLigNPs Characterization by XRD

The crystal structure of the lyophilized AgLigNPs samples was analyzed using X-ray diffraction (XRD) with a PANalytical X’Pert PRO instrument (PANalytical, NL). Powder diffraction data were collected at room temperature with an X’Pert3 θ–θ Powder diffractometer, utilizing Bragg–Brentano parafocusing geometry and Cu Kα radiation (λ = 1.54060 Å, *U* = 40 kV, *I* = 30 mA). Scans were conducted over an angular range of 5°–90° (2θ) with a step size of 0.0390° (2θ) and a counting time of 354.96 s per step. Data collection employed the ultrafast 1D detector PIXCEL. Data evaluation was performed using HighScore Plus 4.0 software (Malvern Panalytical, UK) and referenced against the PDF-4+ database. The crystallite size was determined using the Scherrer equation.

### Characterization by UV–Vis

UV–vis spectroscopy was employed to analyze both the supernatants and isolated AgLigNPs, as well as to determine the stability of AgNPs in various media (LB, TSB, DMEM + 10% FBS, and UPW). For the stability assessment, AgNPs were diluted to a concentration of 10 mg L^–1^ silver content and incubated for 24 h at 37 °C with continuous agitation at 150 rpm using an orbital shaker. The UV–vis spectra were recorded in the range of 300–800 nm, with a 5 nm interval, using a Reader Infinite M900Pro (TECAN MTP, CH).

### Silver Ions Release

The release of silver ions from AgLigNPs and AgNPs was monitored using an ICP-MS ELAN 6000 (PerkinElmer, USA). The nanoparticles were dispersed in various media (LB, TSB, DMEM + 10% FBS, UPW) to achieve a silver concentration of 10 mg L^–1^. A 2 mL sample of each dispersion was placed into a gamma-irradiated dialysis cassette with a molecular weight cutoff (MWCO) of 2,000 and membrane pore size 0.45–0.60 nm (Slide-A-Lyzer G2, Thermo Fisher, USA). The filled cassettes were immersed in 600 mL of UPW equilibrated to 37 °C and placed on an orbital shaker (150 rpm, 37 °C, 48 h). Aliquots of the dialyzate were collected at various time intervals (0.5–48 h) and stabilized with 50 μL of ANALPURE HNO_3_ (ANALYTIKA spol. s.r.o., CZ). Two parallel dialysis runs were performed for each dispersion.

### Culture Media

In this study, three culture media were used: Luria–Bertani (LB), Tryptone Soya Broth (TSB), and high-glucose Dulbecco’s modified Eagle’s Medium (DMEM) supplemented with 10% fetal bovine serum (FBS). The LB medium was composed of 10 g L^–1^ tryptone (Oxoid, UK), 10 g L^–1^ NaCl (Lach:ner, CZ), and 5 g L^–1^ yeast extract (Carl Roth, DE). TSB was used as provided (Oxoid, UK). The high-glucose DMEM and FBS were sourced from Sigma-Aldrich, USA. The LB and TSB media were sterilized by autoclaving. The DMEM and FBS were supplied as sterile by the manufacturer.

### Antibacterial Activity of AgLigNPs against Planktonic and Adhering Cells

In this study, three strains of *P. aeruginosa* (PA) were utilized: ATCC 10145, ATCC 15442, and ATCC BAA-47 (PA01). Prior to antimicrobial testing, LB media were inoculated with PA colonies stored at 4 °C on LB agar plates. The cultures were grown for 24 h at 37 °C with continuous agitation at 150 rpm using an orbital shaker.

The antibacterial efficacy of LigNPs, AgLigNPs, and AgNPs against PA planktonic cells was evaluated using the Bioscreen C microcultivation device (Oy Growth Curves Ab Ltd., FI), following the methodology described by Miškovská et al.^154^ NPs dispersed in water to a final volume of 70 μL were introduced into a microtiter plate to achieve final concentrations of 5–160 mg L^–1^ of lignin in LigNPs or AgLigNPs and 0.3125–320 mg L^–1^ of silver in AgNPs. Subsequently, 210 μL of LB medium and 30 μL of harvested cells (9,000 rcf, 10 °C, 10 min) resuspended in fresh LB medium (OD_600_ = 0.100 ± 0.010) were added to achieve a total volume of 320 μL. Bacterial growth was monitored for 24 h at 37 °C. The growth curves were plotted to determine the minimum inhibitory concentration required to inhibit 50% of the bacterial population (MIC_50_) and the concentration required to inhibit the complete growth of the bacterial population (MIC). Additionally, the minimum bactericidal concentration (MBC)^170^ was determined, representing the concentration that inhibits any visible bacterial growth after transferring 5 μL of treated planktonic cells onto LB agar plates incubated for 24 h at 37 °C. Each experiment was performed in quintuplicate and three independent repetitions.

The antibacterial efficacy of LigNPs, AgLigNPs, and AgNPs against PA-adhering cells was examined using 96-well polystyrene microtiter plates (TPP, CH), following a protocol previously described in Maršík et al.^171^ NPs dispersed in water were introduced into the microtiter plates in a final volume of 70 μL, achieving concentrations of 5–160 mg L^–1^ for lignin in LigNPs or AgLigNPs and 0.3125 to 320 mg L^–1^ for silver in AgNPs. Subsequently, 210 μL of harvested cells (9,000 rcf, 10 °C, 10 min) resuspended in fresh LB medium (OD_600_ = 0.800 ± 0.010) were added to reach a total volume of 280 μL. The plates were incubated for 24 h at 37 °C with continuous agitation at 150 rpm using an orbital shaker. After incubation, the wells were washed twice with sterile PBS (pH 7.4) using an automated microplate washer and dispenser (BioTek 50 TS Washer, USA) to remove nonadherent cells. The metabolic activity of the adhered cells was assessed using the MTT assay, based on the method described by Kulišová et al.,^172^ with slight modifications. The washed wells were filled with 60 μL of glucose solution in PBS (57.4 g L^–1^) and 50 μL of MTT solution in PBS (1.0 g L^–1^) and incubated for 60 min at 37 °C with continuous shaking at 150 rpm. Following incubation, a solvent solution (pH 4.7), consisting of 160 g L^–1^ SDS, 400 g L^–1^ DMF, and 20 g L^–1^ acetic acid in PBS (pH 7.4), was added to dissolve the formazan crystals. The plates were then incubated for an additional 30 min at 37 °C with shaking at 230 rpm. Absorbance readings were taken at 570 nm using a UV–vis spectrophotometer, Infinite M200 Pro reader (Tecan, CH). Each experiment was conducted in octuplicate and three independent repetitions, allowing the determination of the minimum biofilm inhibiting concentration (MBIC_50_), which is the concentration required to reduce biofilm formation by 50% compared to a nanoparticle-free control, which was considered as 100%.

### Cytotoxicity of AgLigNPs

The cytotoxic effects of the prepared nanoparticles were evaluated on two different cell lines—human kidney cells (HEK-293) and human keratinocytes (HaCaT) following an established protocol.^173^ Briefly, cells were plated at a density of 3 × 10^5^ cells mL^–1^ in a 48-well plate using DMEM supplemented with 10% FBS under a 5% CO_2_ atmosphere at 37 °C. The next day, the cells were treated with LigNPs, AgLigNPs, or AgNPs at concentrations ranging from 5 to 160 mg L^–1^ of lignin/lignin/silver, and the incubation continued under the same conditions for another 48 h. Resazurin was then added to each sample at a final concentration of 25 μg mL^–1^, and the cultures were incubated for an additional 4 h. Metabolic activity was assessed using a Microplate Reader Infinite M200 Pro (Tecan, CH) with an excitation at 560 nm and emission at 590 nm. All experiments were performed in triplicate.

For the study of cell morphology under treatment, the same protocol as that for metabolic activity determination was followed, but cells were directly cultured in glass-bottom dishes (MatTek, USA). Before microscopy, the cells were washed three times with PBS and supplemented with 1 mL of fresh DMEM. The cells were visualized using the 3D Cell Explorer (NanoLive, CH). The captured images were processed using Steve v1.6.3496 software (NanoLive, CH).

## Supporting Information Available

Scanning electron microscopy of unincorporated lignin residues separated during the LigNPs isolation process; powder X-ray diffraction pattern of AgLigNPs; UV–vis absorption spectra of LigNPs at a concentration of lignin 87 mg L^–1^, AgLigNPs at a concentration of lignin/silver 87/10 mg L^–1^, AgNPs at a concentration of silver 10 mg L^–1^; light microscopy images of HEK-293 cells: (a) untreated control and (b) cells exposed to LigNPs at a concentration of 160 mg L^–1^ (PDF)
