# Supplementary material for: Preparation and Biological Activity of Lignin–Silver Hybrid Nanoparticles
Source: ACS Omega. 2024 Nov 20;9(48):47765–87. doi: 10.1021/acsomega.4c08117 (PMC11618447; doi:10.1021/acsomega.4c08117)
Supplement: Supplementary file 1 — ao4c08117_si_001.pdf [file ao4c08117_si_001.pdf]

Supporting Information

Preparation and Biological Activity of lignin-silver hybrid nanoparticles

Dominik Maršík[1*], Matěj Danda[1], Jaroslav Otta[2], Petter P. Thoresen[3], Olga Maťátková[1], Ulrika Rova[3], Paul Christakopoulos[3], Leonidas Matsakas[3*], Jan Masák[1]

[1] Department of Biotechnology, University of Chemistry and Technology, 166 28 Prague, Czech Republic

[2] Department of Physics and Measurements, University of Chemistry and Technology, 166 28 Prague, Czech Republic

[3] Biochemical Process Engineering, Division of Chemical Engineering, Department of Civil, Environmental and Natural Resources, Luleå University of Technology, 971 87 Luleå, Sweden

*corresponding authors

Email: marsikd@vscht.cz, leonidas.matsakas@ltu.se

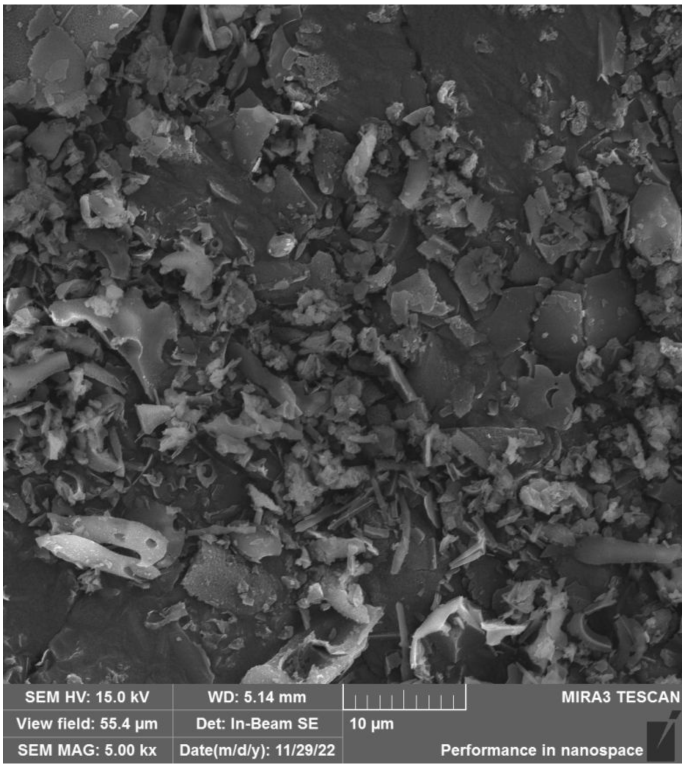

Figure S1 Scanning electron microscopy of unincorporated lignin residues separated during the LigNPs isolation process.

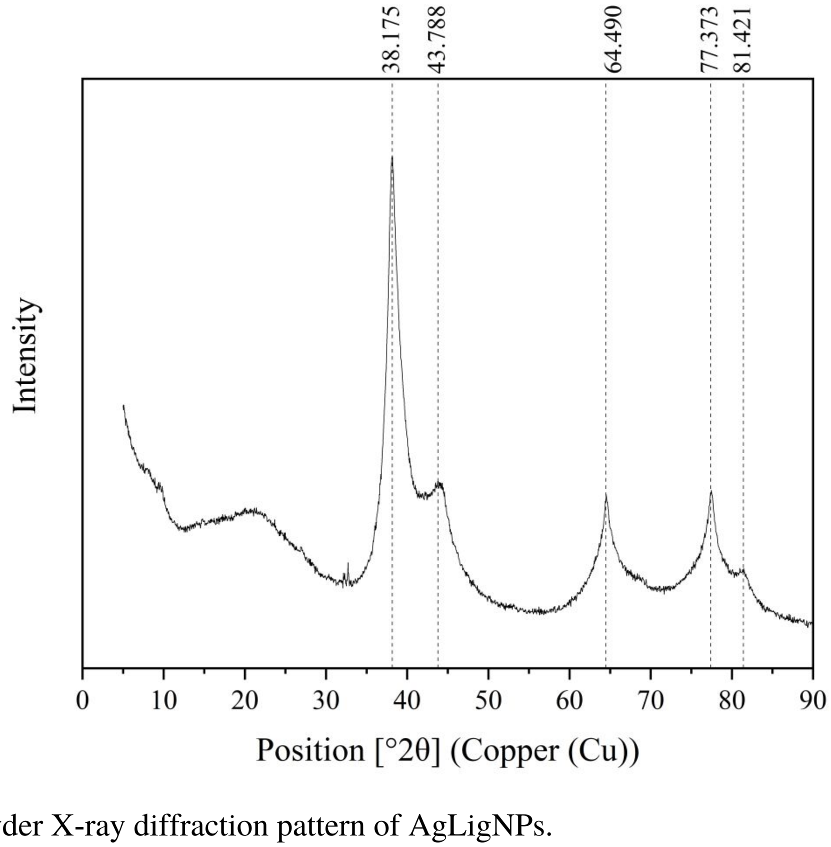

Figure S2 Powder X-ray diffraction pattern of AgLigNPs.

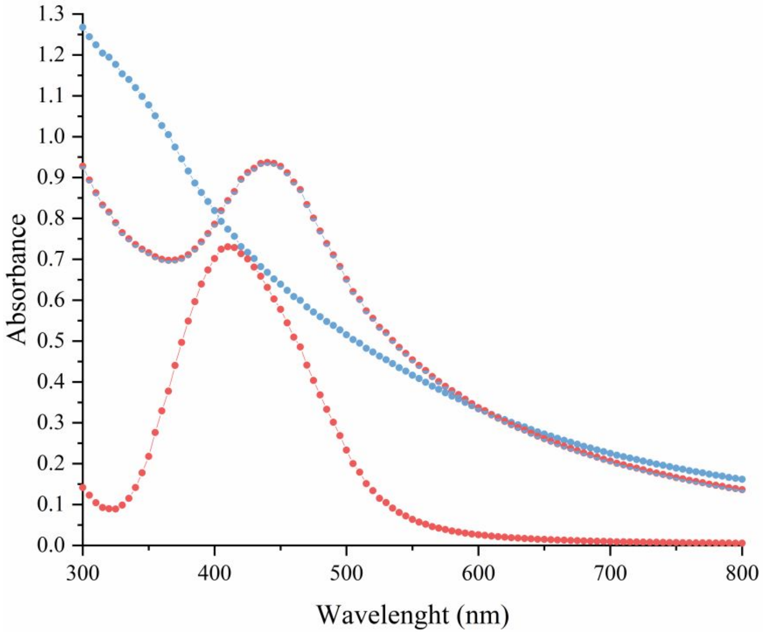

Figure S3. UV-Vis absorption spectra of LigNPs at a concentration of lignin 87 mg L^{-1} ●, AgLigNPs at a concentration of lignin/silver 87/10 mg L^{-1} ●, AgNPs at a concentration of silver 10 mg L^{-1} ●.

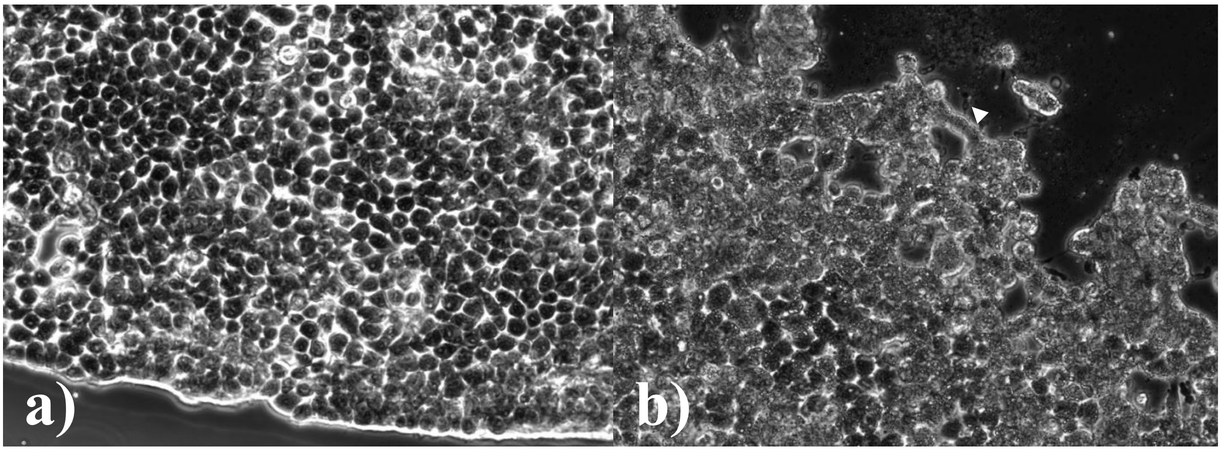

Figure S4 Light microscopy images of HEK-293 cells a) untreated control and b) cells exposed to LigNPs at a concentration of 160 mg L^{-1}. The white arrowhead indicates the presence of apoptotic bodies.
